# Morpho-cultural, pathogenic, and molecular diversity, and population structure analysis of *Bipolaris sorokiniana* causing spot blotch disease in wheat

**DOI:** 10.3389/fmicb.2026.1811921

**Published:** 2026-06-17

**Authors:** Ankush Kumar, Ravindra Kumar, Rajender Singh, Chandra Nath Mishra, Binay K. Singh, Charan Singh, Shyam Saran Vaish, Shiv Shankar Patel, Arun Gupta, Ratan Tiwari

**Affiliations:** 1 Department of Plant Pathology, CCS Haryana Agricultural University, Hisar, Haryana, India; 2 ICAR-Indian Institute of Wheat and Barley Research, Karnal, Haryana, India; 3 Department of Mycology and Plant Pathology, Institute of Agricultural Sciences, Banaras Hindu University, Varanasi, Uttar Pradesh, India

**Keywords:** *Bipolaris sorokiniana*, pathogenic variability, population structure, spot blotch, SSR markers, wheat

## Abstract

Spot blotch, incited by *Bipolaris sorokiniana,* is one of the serious foliar diseases affecting wheat productivity worldwide, significantly. The cultural, morphological, pathogenic, and molecular variability were studied comprehensively among 51 *B. sorokiniana* isolates collected from diverse wheat-growing regions of India. Regarding cultural variability, the highest radial growth rate was observed in isolate Bs-9 (8.45 mm/day), whereas isolate Bs-27 exhibited the lowest growth rate (2.50 mm/day). Morphological analysis indicated that isolate Bs-3 had the greatest conidial length, conidial breadth, and number of septa, whereas isolate Bs-12 displayed the minimum values for these traits. A highly significant positive correlation was observed among conidial length, conidial breadth, and number of septa. Pathogenic variability analysis identified isolate Bs-3 (Manikchak, West Bengal) as the most virulent, exhibiting the shortest incubation period (2.75 days), the largest mean lesion length (5.05 mm) and lesion breadth (1.69 mm), maximum lesion size (8.53 mm^2^), lesion coverage (67.50%), and the highest disease score (4.5). A highly significant negative correlation (*r* = −0.87**, *p* < 0.001) was observed between incubation period and disease score. Population structure analysis using 28 SSR markers separated the isolates into two distinct sub-populations (SP1 and SP2). Analysis of molecular variance (AMOVA) indicated that 71% of the total variation was due to differences within subpopulations, whereas 29% was attributed to variation between subpopulations. The mean number of effective alleles (*Ne*) was 7.118 for SP1 and 8.333 for SP2. Shannon’s information index (*I*) was higher in SP2 (2.238) than in SP1 (2.011). SP2 also exhibited greater genetic diversity, as reflected by higher expected heterozygosity (*He* = 0.880) and unbiased heterozygosity (*uH* = 0.917) compared to SP1. The results demonstrated significant variation among *B. sorokiniana* isolates in morphological, cultural, pathogenic, and molecular attributes. Notably, isolate Bs-3 was identified as a highly virulent strain and may serve as a reference isolate for rigorous screening of wheat germplasm and for detailed studies of wheat- *B. sorokiniana* interactions.

## 1 Introduction

Wheat (*Triticum aestivum* L.), belonging to the family Poaceae, represents one of the most important cereal crops globally. It is the principal staple food for more than one-third of the world’s population (Roy et al., 2023). The evolutionary history of wheat involves a complex sequence of hybridization and polyploidization, beginning with diploid einkorn wheat (*T. monococcum*, 2n = 2x = 14, AA), progressing to tetraploid emmer wheat (*T. turgidum*, 2n = 4x = 28, AABB), and culminating in hexaploid bread wheat (*T. aestivum*, 2n = 6x = 42, AABBDD) through the introgression of the D genome from *Aegilops tauschii* (Sax and Sax, 1924; Watkins, 1930; Kimber and Sears, 1987; Avni et al., 2017). At present, bread wheat accounts for nearly 95% of total global wheat production, whereas durum and dicoccum wheats contribute approximately 4 and 1%, respectively (Grewal and Goel, 2015).

Since its domestication in the Fertile Crescent over 8,000 years ago, wheat has played a crucial role in human nutrition and food security, particularly in Europe, South Asia, and North Africa. Globally, wheat is cultivated on 220.4 million hectares, yielding an annual production of approximately 799 million tonnes (FAOSTAT, 2023). In India, it is the second most important cereal crop after rice, covering 31.83 million hectares, producing 113.29 million tonnes, and having an average productivity of 3.56 tonnes per hectare (DOA & FW, 2024). Wheat contributes about 20% of dietary calories and 25% of protein intake in the human diet, and serves as a significant source of carbohydrates, minerals such as iron and zinc, and vitamins, particularly thiamine (Kohajdová and Karovicova, 2008; Remi and Esther, 2025).

Although significant genetic gains in productivity have been achieved through modern breeding and agronomic practices, wheat production remains susceptible to both biotic and abiotic stresses. Among biotic stresses, foliar diseases represent a persistent challenge to sustainable wheat cultivation globally, resulting in annual yield losses of approximately 20% (Devi et al., 2022). Specifically, spot blotch, caused by the hemibiotrophic fungus *B. sorokiniana* is considered as one of the most destructive wheat diseases in warm, humid regions (Figueroa et al., 2018; Gupta et al., 2018; Raj et al., 2024).

Spot blotch was first documented in 1914 by Mohy (Joshi et al., 2004) and has since become prevalent in major wheat-producing regions, including South and Southeast Asia, North America, Latin America, Australia, and parts of Africa (Joshi et al., 2007a; Kumar et al., 2020). The disease initially manifests as small, light-brown, elliptical-to-oval lesions with chlorotic halos on leaves, sheaths, glumes, and nodes. These lesions subsequently enlarge, merge, and induce premature leaf senescence (Gupta et al., 2018; Kumar et al., 2020). Under favorable conditions, specifically temperatures of 25–30 °C, high relative humidity, and extended leaf wetness (12–18 h), the pathogen proliferates rapidly, resulting in yield losses of up to 70% and, in severe epidemics, complete crop failure (Sharma and Duveiller, 2003; Pandey et al., 2008; Al-Sadi, 2021; Roy et al., 2023). Infected seed acts as the primary source of inoculum, while wind-dispersed conidia contribute to secondary spread. The pathogen can survive in infected crop residues and in soil in the absence of a suitable host, thereby facilitating its persistence between growing seasons (Pandey et al., 2008).

Spot blotch has become highly prevalent in South Asia, particularly in the Indo-Gangetic Plains of India, Bangladesh, and Nepal, and now severely constrains wheat productivity (Sharma and Duveiller, 2006; Joshi et al., 2007a; Gupta et al., 2018; Kumar et al., 2024). India accounts for nearly 40% of the global 25 million hectares of wheat affected by this disease (Joshi et al., 2007a, 2007b). Notably, spot blotch is expanding into cooler regions such as the North Western Plain Zone (NWPZ) of India, which was traditionally considered as a low-disease zone. Recent survey-based studies have highlighted its widespread occurrence and increasing importance under changing climatic conditions, with significant variability in disease incidence across regions (Özer et al., 2020; Devi et al., 2021; Roy et al., 2023; Chakraborty et al., 2024; Yang et al., 2024). These findings underscore the need for detailed investigations into pathogen variability and population structure to support effective disease management strategies. This trend indicates the increasing adaptability of the pathogen to changing climatic conditions (Singh et al., 2013; Singh et al., 2020). As a result, spot blotch has shifted from a regional issue to a nationally important disease in India, presenting a major challenge to wheat improvement programs.

The *B. sorokiniana* population exhibits considerable genetic variability, as demonstrated by distinct cultural, morphological, pathogenic, and molecular characteristics (Misra, 1981; Maraite et al., 1998; Momtaz et al., 2019; Verma et al., 2020; Chakraborty et al., 2024). Characterizing this variability is essential for developing durable, broad-spectrum resistance in wheat cultivars. While previous studies have documented regional variability using either morphological or molecular methods, comprehensive assessments integrating morpho-cultural, pathogenic, and molecular diversity across multiple agro-climatic zones in India are still scarce. Molecular techniques, particularly simple sequence repeat (SSR) markers, provide robust tools for analyzing population structure, genetic differentiation, and evolutionary dynamics of fungal pathogens (Fajolu et al., 2013; Chauhan et al., 2017; Raja et al., 2017). These analyses are vital for identifying virulent lineages, monitoring pathogen evolution, and informing targeted breeding strategies. In this regard, this study aimed to (1) collect and characterize *B. sorokiniana* isolates from various wheat-growing regions of India, (2) assess the morpho-cultural, pathogenic, and molecular diversity among these isolates using SSR markers, and (3) elucidate the population structure and genetic relationships among isolates. The findings offer a comprehensive understanding of the population biology and diversity of *B. sorokiniana*, thereby supporting the development of effective disease management strategies and durable resistance breeding in wheat. Previous studies, including recent work on *Cochliobolus* anamorphs (Özer et al., 2020) have primarily focused on species identification and interspecific variability using molecular markers. However, limited attention given to linking intraspecific genetic structure with pathogenic variability in *B. sorokiniana* populations across diverse agro-climatic regions. The present study addresses this gap by integrating phenotypic, pathogenic, and population genetic analyses. For instance, recent investigations on *Cochliobolus* anamorphs and *Bipolaris* spp. have highlighted the importance of multilocus approaches and polymorphic markers in resolving intraspecific variability and linking genetic structure with pathogenic behavior (Özer et al., 2020; Chakraborty et al., 2024; Yang et al., 2024). However, such integrative studies remain limited for *Bipolaris sorokiniana* populations across diverse agro-climatic regions of India. Therefore, the present study aims to bridge this gap by combining morpho-cultural, pathogenic, and SSR-based genetic analyses to better understand variability and its implications for disease management and resistance breeding.

## 2 Materials and methods

### 2.1 Collection, isolation, and purification of *B. Sorokiniana* isolates

**Figure 1:**
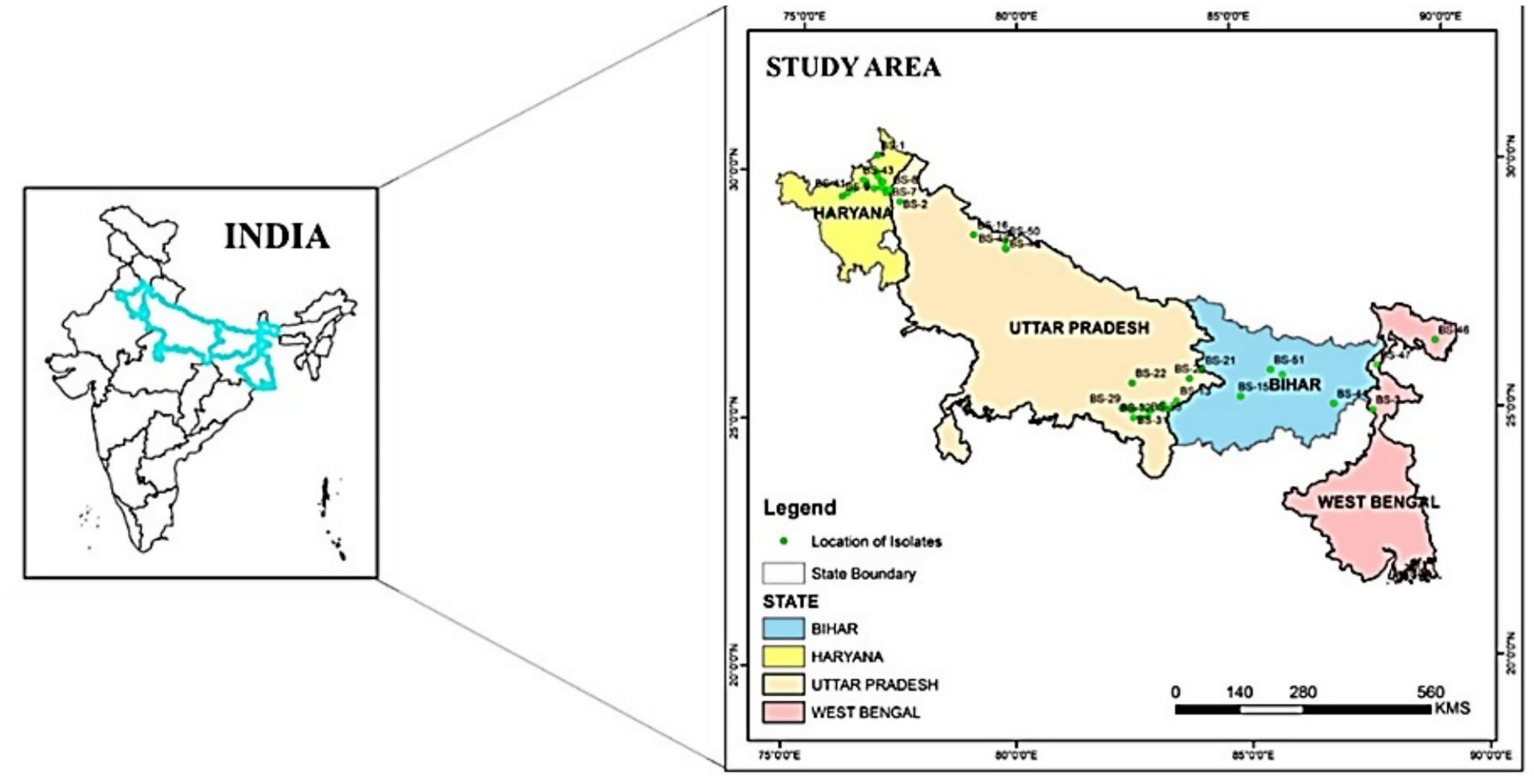
Geographic locations of spot blotch–infected wheat sample collections.

During the 2022–23 *Rabi* season, roving surveys were conducted in major wheat-growing districts of Haryana and several districts of Uttar Pradesh to collect leaf samples infected with spot blotch disease. Additional infected samples were obtained from Bihar and West Bengal. All *B. sorokiniana* isolates collected from these locations were designated as Bs-1 to Bs-51 (Supplementary Table 1 and Figure 1). The pathogen was isolated using the method described by Chand et al. (2013). Spot blotch infected leaf samples were washed in running tap water and cut into small bits (2–3 mm) by using sharp sterilized scalpel blade. The bits were surface sterilized with 0.1% mercuric chloride (HgCl_2_) for 15 to 30 s and rinsed with sterilized water for 2 to 3 times serially for removing the residual traces of mercuric chloride from bits. The surface sterilized bits transferred to Petri plates containing sterilized Potato Dextrose Agar (PDA) medium. Potato Dextrose Agar (PDA) medium was prepared using 200gm of potato slices boiled in 500 mL of distilled water, the solution was filtered through a piece of muslin cloth. In another conical flask, 20 g of Dextrose and 20 g of Agar were boiled in 500 mL of distilled water. We mixed all the solutions and made the volume up to 1,000 mL, followed by sterilization of the medium in an autoclave at 120 °C and 15 psi for 15–20 min. A pinch of streptomycin was also incorporated into medium during its pouring to circumvent, bacterial contaminations in culture. Inoculated Petri plates were incubated at 28 ± 1 °C in BOD incubator. The incubated Petri plates were observed after 24 h for presence of fungal growth. The fresh growth was transferred on fresh PDA plates to obtain pure culture of each isolate. On solidification, single conidium was located under inverted position and marked by glass marking pencil. Single conidium was transferred aseptically to PDA slants and incubated at 28 ± 1 °C. The culture obtained from single conidia was pure and used throughout the course of investigation (Kumar et al., 2007).

### 2.2 Identification and maintenance of pathogen isolates

The identity of *B. sorokiniana* isolates was confirmed by preparing slides and examining them under a phase-contrast microscope (Carl Zeiss Axio Imager M2, Germany) to assess morphological characteristics, including conidial size and septation. In addition to microscopic analysis, cultural characteristics, including colony growth, texture, color, margins, and zonation, were recorded. Identification was done according to the method described by Manamgoda et al. (2014). Final confirmation was made on basis of ITS (590–600 bp) band. Purified cultures were maintained on slants at 5 °C in a refrigerator, with sub culturing performed every 6 to 8 months.

### 2.3 Mass multiplication of *B. sorokiniana* inoculum and inoculation for pathogenicity study

Isolates of *B. sorokiniana* were mass multiplied according to the procedure outlined by Chand et al. (2013). Sorghum grains were soaked in water for 24 h, and excess water was removed using a muslin cloth. The moistened grains were transferred into 150 mL volumetric flasks, filling one-third of the flask volume, and sterilized by autoclaving at 121.6 °C for 20 min. Sterilized grains were inoculated with freshly grown cultures of each *B. sorokiniana* isolate. After 4 to 5 days of incubation, cultures were gently shaken to promote uniform sporulation and then incubated for an additional 10 to 15 days at 28 ± 1 °C. A spore suspension (10^4^ spores mL^−1^) was prepared by soaking colonized sorghum grains in distilled water, and 100 μL of Tween 20 was added per litre of suspension to ensure even dispersion. Inoculation was performed by uniformly spraying the spore suspension onto plants at the two-leaf stage, following the method described by Mahapatra and Das (2011) for pathogenic variability studies.

### 2.4 Cultural and morphological characterization

The cultural characteristics of *B. sorokiniana* isolates were assessed on potato dextrose agar (PDA) medium following the methodology outlined by Chauhan et al. (2017), Verma et al. (2020), and Chakraborty et al. (2024). Evaluated traits included colony color, margin, radial growth, texture, and exudation, with observations made 10 days after inoculation. Radial growth was measured by placing a 5 mm mycelial disc from the actively growing margin of each isolate at the center of 90 mm PDA plates. Each isolate was tested in triplicate and incubated at 28 ± 1 °C. Colony diameters were recorded on the 3rd, 5th, 7th, and 10th days along the two perpendicular axes. Growth rate was calculated as daily radial expansion to facilitate comparison among isolates. Colony color was determined using the Munsell color chart (Munsell, 1905), and additional cultural traits, such as texture, appearance, margin, and exudation, were documented 10 days post-inoculation (Prasad et al., 2012). Morphological variability was evaluated based on conidial characteristics, including length, breadth, and septation. Isolates were clustered based on combined morphological and cultural traits using a dendrogram constructed from Euclidean squared distances and Ward’s minimum-variance method (Jobson, 1992). Microscopic images were obtained using an Axiom 503 mono camera, and precise measurements of conidial length, breadth, and septa number were recorded for each isolate.

### 2.5 Pathogenic characterization

Pathogenic variability among *B. sorokiniana* isolates was assessed under controlled artificial inoculation conditions in a polyhouse at the ICAR–Indian Institute of Wheat and Barley Research, Karnal, India. The wheat cultivar ‘Sonalika’ served as the susceptible check, while ‘Chirya-3’ was used as the resistant cultivar for spot blotch. These cultivars were evaluated to quantify their responses to infection by various *B. sorokiniana* isolates. Seeds were sown in 12 cm diameter pots filled with nutrient-rich soil in the polyhouse. Each treatment was replicated five times, with each pot representing a replicate and containing three plants. At the two-leaf stage, leaves were inoculated with a spore suspension using a hand sprayer until run-off (Mahapatra and Das, 2011). After inoculation, humidity was maintained to facilitate pathogen growth and disease development. The incubation period was recorded daily following inoculation. Lesion-related parameters, including lesion length, lesion breadth, lesion size, infection frequency (number of lesions per cm^2^ leaf area), lesion cover, and disease severity, were measured 20 days after inoculation, following the methods described by Chauhan et al. (2017) and Sultana et al. (2018). Disease severity was rated on a 1–5 scale according to Rahman et al. (2011), where 1 indicates no visible symptoms or chlorotic flecks, 2 indicates up to 10% leaf area covered with small restricted lesions, 3 indicates 11–25% leaf area covered with small restricted lesions, 4 indicates 26–50% leaf area covered with large coalescing lesions, and 5 indicates more than 50% leaf area covered with large coalescing lesions. Disease assessment was conducted from 3 to 20 days after inoculation, and the Percent Disease Index (PDI) was calculated using the formula proposed by McKinney (1923). A dendrogram was constructed to cluster the isolates based on their pathogenic traits using Euclidean squared distance and Ward’s minimum variance method (Jobson, 1992).

### 2.6 Molecular characterization

Genetic variability among *B. sorokiniana* isolates was evaluated using SSR markers. Mycelial mats were generated by inoculating 5 mm discs from 7-days-old, actively growing cultures into 100 mL of potato dextrose broth (PDB) medium in 250 mL conical flasks. Flasks were sterilized at 121.6 °C and 15 psi for 15 min, and then incubated at 28 ± 1 °C for 15 days in a BOD incubator shaker. The resulting mycelium aseptically filtered through Whatman No. 1 filter paper, dried, wrapped in aluminium foil, and stored at −20 °C. Genomic DNA was extracted using a modified CTAB protocol (Kumar et al., 2013). DNA quantity and quality were determined by measuring the A260/A280 absorbance ratio using a NanoQuant Infinite® 200 spectrophotometer (TECAN) and by agarose gel electrophoresis. SSR primers associated with *B. sorokiniana* diversity were standardized for annealing temperature using a representative DNA sample, and polymerase chain reaction (PCR) amplification was performed under optimized conditions (Table 1). Universal internal transcribed spacer (ITS) primers ITS1 (5′-CTTGGTCATTTAGAGGAAGTAA-3′) and ITS4 (5′-TCCTCCGCTTATTGATATGC-3′) (White et al., 1990) were also used to confirm isolate identity based on the conserved ITS region. Each PCR reaction (25 μL) contained 12.5 μL of PCR master mix, 7.5 μL of sterile double-distilled water, 3 μL of template DNA, and 1 μL each of forward and reverse primers. Thermal cycling conditions included an initial denaturation at 94 °C for 2 min, followed by 35 cycles of 94 °C for 45 s, 59 °C for 60 s, and 72 °C for 90 s, with a final extension at 72 °C for 10 min. Amplified products were separated on 2.5% agarose gels in 1 × TBE buffer, stained with ethidium bromide, and visualized using a Bio-Rad gel documentation system. Fragment sizes were estimated using a 100 bp DNA ladder. Gels were scored for band presence or absence to generate molecular profiles for each isolate.

**Table 1:** Genetic characteristics of 28 polymorphic simple sequence repeat (SSR) loci in 48 *Bipolaris sorokiniana* isolates.

**Locus**	**Forward primer sequence (5′-3′)**	**Reverse primer sequence (5′-3′)**	**Tm (°C)**	**Allele size (bp)**	**MAF**	**Genetic diversity**	**PIC**
BS-4	GTTCTTGTTCTGCAGGTGCG	GAACAGCAGAGAAAAGGCG	60	214	0.6458	0.4575	0.3528
BS-6	GATTTTGATCGAAGGGGCGG	ACCTCATATGCGCACAAAAG G	59	157	0.9167	0.1528	0.1411
BS-6_1	GATTTTGATCGAAGGGGCGG	ACCTCATATGCGCACAAAAG G	59	165	0.6667	0.4444	0.3457
BS-7	TGGATTTGTCGGAGTTGAATTG C	TTTCCAACGGAAATTCGCGG	60	199	0.7708	0.3533	0.2909
BS-9	CGGTTAGCCACAGCAAAGC	TGTATTGTTCAAGCTGGCGC	59	153	0.9375	0.1172	0.1103
BS-9_1	CGGTTAGCCACAGCAAAGC	TGTATTGTTCAAGCTGGCGC	59	160	0.9167	0.1528	0.1411
BS-10	CAACATGCTCGTTACCGTGG	TCACGCATCTAAGCAGCAGC	59	125	0.9583	0.0799	0.0767
BS-11	GGAACCTACTCCGACGTTGC	ATGTACAGACGCACGTCAGC	60	209	0.5417	0.4965	0.3733
BS-12	ACGGGTAAATCATCGGTGCC	TGGTGCAGGTATGAAGACGG	60	175	0.75	0.375	0.3047
BS-12_1	ACGGGTAAATCATCGGTGCC	TGGTGCAGGTATGAAGACGG	60	190	0.7708	0.3533	0.2909
BS-13	TTGCTGCTGCCTTGTATTGC	GCGTGCTGCAACAATGGG	59	130	0.7083	0.4132	0.3278
BS-14	ACGAGTCCTTTTTACCACAGC	ATCTGGCGTACTTTCCGTCC	58	177	0.6875	0.4297	0.3374
BS-14_1	ACGAGTCCTTTTTACCACAGC	ATCTGGCGTACTTTCCGTCC	58	190	0.9375	0.1172	0.1103
BS-18	TCCACCCCAATTCTATACTTACTACC	TAATCAGAGGGGCAAAGGG C	59	209	0.7708	0.3533	0.2909
BS-20	TTGCCCATTGCTCGTTACCC	GAGGGGTTTCAGCAGTAGGG	60	183	0.6458	0.4575	0.3528
BS-21	AGGCTGAAGCTGACAAAGGC	TTGGAGGAGAAGGAGGACG G	60	182	0.9375	0.1172	0.1103
BS-21_1	AGGCTGAAGCTGACAAAGGC	TTGGAGGAGAAGGAGGACG G	60	200	0.8333	0.2778	0.2392
BS-24	ATGTGGGAATACGGGGAAGG	TTCAGCCAAGTCTCTTGTGC	59	205	0.5208	0.4991	0.3746
BS-24_1	ATGTGGGAATACGGGGAAGG	TTCAGCCAAGTCTCTTGTGC	59	220	0.9583	0.0799	0.0767
BS-25	AGACCATCTGTTGCCCAACC	CAGACTGATTCCTTGTCGAG C	60	165	0.7917	0.3299	0.2755
BS-25_1	AGACCATCTGTTGCCCAACC	CAGACTGATTCCTTGTCGAG C	60	190	0.6042	0.4783	0.3639
BS-26	GCGTTTGCTTTCGATCGTCC	AGGCTGGAGAGGAGAGTTG G	60	157	0.5833	0.4861	0.3680
BS-27	AGACATTGAGGCAGTCGTGG	GGAAAACAGGCCGTTGTTGC	60	148	0.6667	0.4444	0.3457
BS-27_1	AGACATTGAGGCAGTCGTGG	GGAAAACAGGCCGTTGTTGC	60	200	0.75	0.375	0.3047
BS-28	GACATCGTATCTGCCGTGGG	AAAGCTGTCAAATTGCGGGC	60	174	0.5208	0.4991	0.3746
BS-28_1	GACATCGTATCTGCCGTGGG	AAAGCTGTCAAATTGCGGGC	60	185	0.9792	0.0408	0.0400
BS-29	TCAAATGCAATGTATTCTCTACCCG	CACGTCCCATAACGGATTGC	59	159	0.7083	0.4132	0.3278
BS-30	ACAACCTGCCACTATCACGG	CCTAGTGGATGGGCAATGGG	60	169	0.5833	0.4861	0.3680
BS-30_1	ACAACCTGCCACTATCACGG	CCTAGTGGATGGGCAATGGG	60	150	0.9792	0.0408	0.0400
BS-32	TTTTCTTTCTCTCCGCACGC	GTCTTGGGGGTGGACAAGG	59	168	0.625	0.4688	0.3589
BS-34	GAATAGGGAGTGGACGAGAGC	ACAAACGCTGCGTAGATTTC C	59	196	0.8125	0.3047	0.2583
BS-35	GATTGGGCCAGTTGAAACCG	TGCCACCCTCCTCTACTACC	59	188	0.7292	0.395	0.3170
BS-35_1	GATTGGGCCAGTTGAAACCG	TGCCACCCTCCTCTACTACC	59	200	0.9792	0.0408	0.0400
BS-36	CTGGTAGCGGTAGTGGTAGC	CTTGTAGAGAGGAGCCCTGC	59	158	0.6042	0.4783	0.3639
BS-36_1	CTGGTAGCGGTAGTGGTAGC	CTTGTAGAGAGGAGCCCTGC	59	130	0.9167	0.1528	0.1411
BS-38	CATCAGCCAAACCGTTGACG	TGTACTCTACACGGATGCAT ACG	60	176	0.8542	0.2491	0.2181
BS-39	GCCCCTAGATGAGAACTCGC	GCGAAATTTGCTGCAATCCC	59	178	0.5625	0.4922	0.3711
BS-39_1	GCCCCTAGATGAGAACTCGC	GCGAAATTTGCTGCAATCCC	59	150	0.9792	0.0408	0.0400
BS-40	TCAGTATCTAGTGCGCACCC	TGTGCATTGTTGTGCTGTCC	59	171	0.9583	0.0799	0.0767
BS-40_1	TCAGTATCTAGTGCGCACCC	TGTGCATTGTTGTGCTGTCC	59	150	0.7917	0.3299	0.2755
BS070	AAACAAGAATGCTCCGAAGTTG	CCCGTCCTCATTACCCAGTAT	60	219–265	0.9792	0.0408	0.0400
BS070_1	AAACAAGAATGCTCCGAAGTTG	CCCGTCCTCATTACCCAGTAT	60	205	0.875	0.2188	0.1948
BS074	ACGTAAGGAAAAACACCTCGAGTC	ACTTTATCCGTGTGCATCTTCAAC	66	161–202	0.875	0.2188	0.1948

### 2.7 Analysis of population structure, AMOVA, and genetic diversity indices

Bayesian inference of population structure was conducted using STRUCTURE v2.3.4 (Pritchard et al., 2000) under an admixture model with correlated allele frequencies. Each STRUCTURE analysis included a burn-in period of 100,000 iterations and 500,000 Markov Chain Monte Carlo (MCMC) iterations, with 10 independent replicates for each assumed number of populations (K) from 1 to 10. The optimal number of genetic clusters was identified using the ΔK method of Evanno et al. (2005), implemented via STRUCTURE HARVESTER. Analysis of molecular variance (AMOVA) and Nei’s genetic distance were calculated using the number of subpopulations identified by STRUCTURE (Excoffier et al., 1992) to estimate genetic differentiation among and within subpopulations of *B. sorokiniana* isolates (Nei, 1972). Genetic diversity indices, including the number of different alleles (*Na*), number of effective alleles (*Ne*), observed heterozygosity (*Ho*), expected heterozygosity (*He*), and Shannon’s information index (*I*), were calculated. Population pairwise *PhiPT* values, as an analogue to *Fst*, were also determined. All calculations were conducted in GenAlEx v6.5 using 9,999 permutations (Peakall and Smouse, 2012).

### 2.8 Statistical analysis

The recorded data were analyzed using the R statistical programming environment. Duncan’s Multiple Range Test (DMRT) was applied to assess significance at the 0.05 level. Additional analyses, including Principal Component Analysis (PCA), were conducted in R. Pearson’s correlation coefficients for various traits were calculated in R, and correlation plots were generated using the ‘corrplot’ package. Molecular data analysis was performed using software tools such as Darwin, GenAlEx V6.5, and STRUCTURE.

## 3 Results

### 3.1 Cultural variability

**Figure 2:**
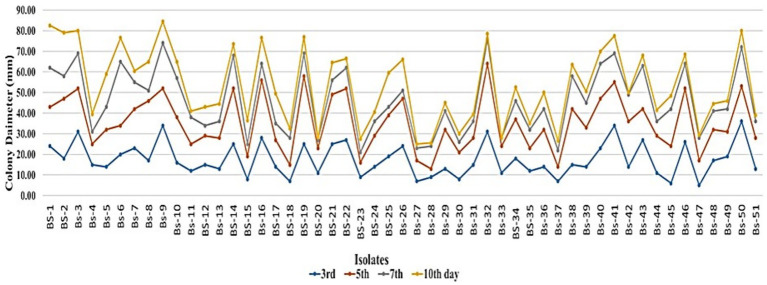
Radial growth of 51 *Bipolaris sorokiniana* isolates measured on PDA medium at 3, 5, 7, and 10 days after inoculation.

**PLATE 1:**
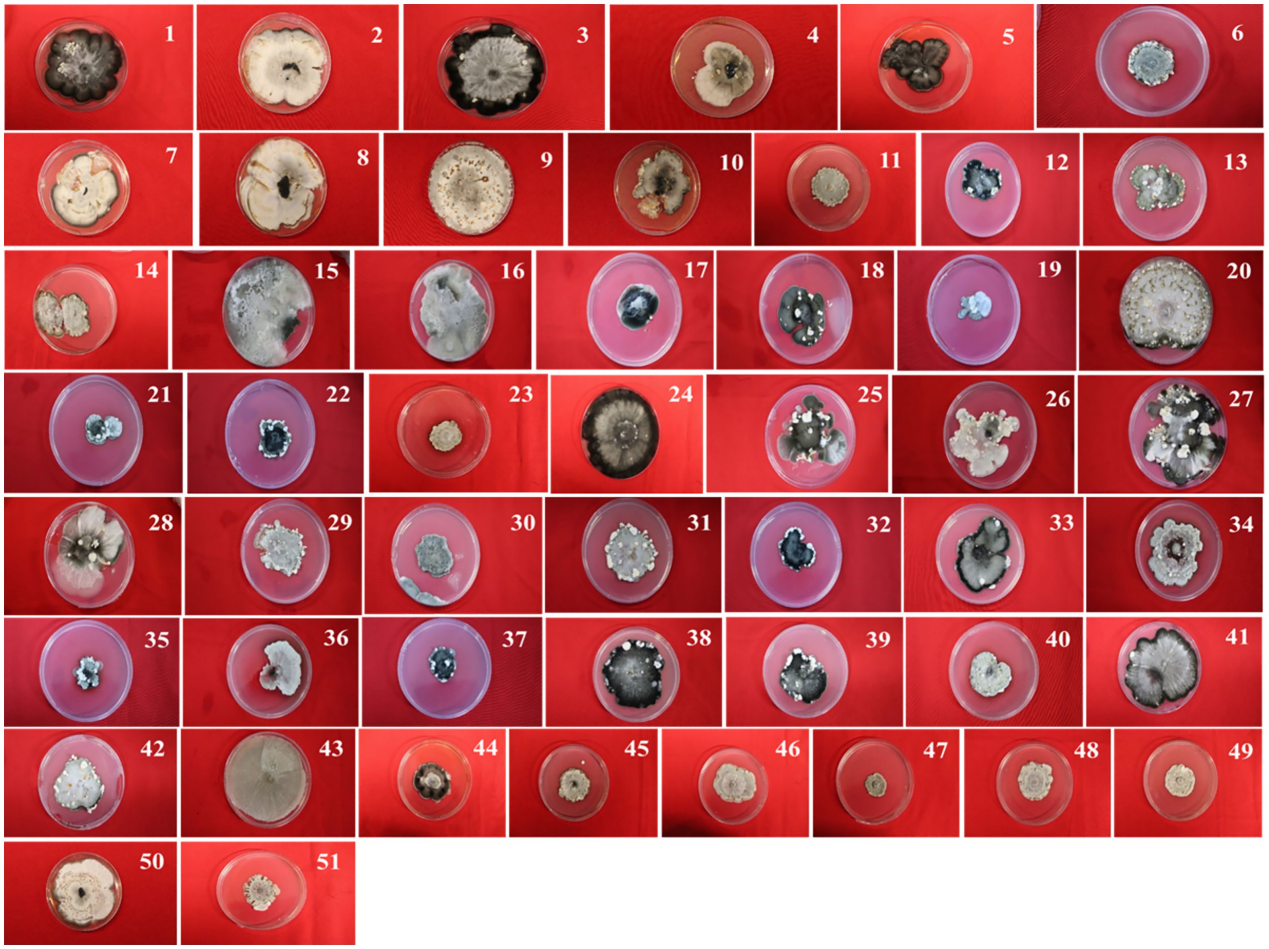
Radial growth and colony characteristics of *Bipolaris sorokiniana* isolates. Numbers 1–51 correspond to isolates Bs-1 to Bs-51.

Fifty-one *B. sorokiniana* isolates demonstrated a considerable level of cultural variation on potato dextrose agar (PDA) medium (Tables 2, 3, Figure 2, and Plate 1). Significant differences in radial growth were observed among isolates at each time point. At 10 days after incubation, colony diameters ranged from 25.0 to 84.5 mm, corresponding to growth rates of 2.50–8.45 mm per day. The fastest-growing isolates included Bs-9 (84.5 mm; 8.45 mm per day), Bs-1 (82.5 mm; 8.25 mm per day), and both Bs-3 and Bs-50 (80.0 mm; 8.0 mm per day), while Bs-27 (25.0 mm; 2.50 mm per day) exhibited the slowest growth. Growth profiles measured at 3, 5, 7, and 10 days consistently diverged, reflecting stable differences in colony expansion among isolates.

**Table 2:** Radial growth and daily growth rate of *Bipolaris sorokiniana* isolates.

**S. No.**	**Isolate**	**Days after inoculation**				**Growth/day (mm)**
		3rd day (mm)*	5th day (mm)*	7th day (mm)*	10th day (mm)*	
1	Bs-1	24^h^	43^j^	62^i^	82.5^b^	8.25^b^
2	Bs-2	18^l^	47^h^	58^j^	79^d^	7.90^d^
3	Bs-3	31^c^	52^f^	69^d^	80^c^	8.00^c^
4	Bs-4	15^o^	25^w^	31^z^	39.5^G^	3.95^G^
5	Bs-5	14^p^	32^r^	43^r^	59^t^	5.90^t^
6	Bs-6	20^j^	34^p^	65^f^	76.5^h^	7.65^h^
7	Bs-7	23^i^	42^k^	55^m^	60.5^r^	6.05^r^
8	Bs-8	17^m^	46^i^	51^n^	65^o^	6.50^o^
9	Bs-9	34^b^	52^f^	74^b^	84.5^a^	8.45^a^
10	Bs-10	16^n^	38^m^	57^k^	65^o^	6.50^o^
11	Bs-11	12^r^	25^w^	38^u^	41^E^	4.10^E^
12	Bs-12	15^o^	29^t^	34^x^	43^C^	4.30^C^
13	Bs-13	13^q^	28^u^	36^v^	44.5^B^	4.45^B^
14	Bs-14	25^g^	52^f^	68^e^	73.5^i^	7.35^i^
15	Bs-15	8^u^	19^A^	25^D^	36.5^I^	3.65^I^
16	Bs-16	28^d^	56^c^	64^g^	76.5^h^	7.65^h^
17	Bs-17	14^p^	27^v^	35^w^	49.5^x^	4.95^x^
18	Bs-18	7^v^	15^D^	28^A^	32.5^K^	3.25^K^
19	Bs-19	25^g^	58^b^	69^d^	77^g^	7.70^g^
20	Bs-20	11^s^	23^y^	27^B^	28.5^N^	2.85^N^
21	Bs-21	25^g^	49^g^	56^l^	64.5^p^	6.45^p^
22	Bs-22	27^e^	52^f^	62^i^	66.5^m^	6.65^m^
23	Bs-23	9^t^	16^C^	21^H^	27.5^P^	2.75^p^
24	Bs-24	14^p^	29^t^	36^v^	40.5^F^	4.05^F^
25	Bs-25	19^k^	39^l^	43^r^	59.5^s^	5.95^s^
26	Bs-26	24^h^	47^h^	51^n^	66^n^	6.60^n^
27	Bs-27	7^v^	17^B^	23^F^	25^R^	2.50^R^
28	Bs-28	9^t^	13^F^	24^E^	25.5^R^	2.55^R^
29	Bs-29	13^q^	32^r^	41^t^	45^A^	4.50^A^
30	Bs-30	8^u^	21^z^	26^C^	30^L^	3.00^L^
31	Bs-31	15^o^	28^u^	36^v^	39.5^G^	3.95^G^
32	Bs-32	31^c^	64^a^	76^a^	78.5^e^	7.85^e^
33	Bs-33	11^s^	24^x^	28^A^	28^O^	2.80^o^
34	Bs-34	18^l^	37^n^	46^p^	52.5^u^	5.25^u^
35	Bs-35	12^r^	23^y^	32^y^	35^J^	3.50^j^
36	Bs-36	14^p^	32^r^	42^s^	50^w^	5.00^w^
37	Bs-37	7^v^	14^E^	22^G^	26.5^Q^	2.65^Q^
38	Bs-38	15^o^	42^k^	58^j^	63.5^q^	6.35^q^
39	Bs-39	14^p^	33^q^	45^q^	50.5^v^	5.05^v^
40	Bs-40	23^i^	47^h^	64^g^	70^j^	7.00^j^
41	Bs-41	34^b^	55^d^	69^d^	77.5^f^	7.75^f^
42	Bs-42	14^p^	36^o^	49^o^	50^w^	5.00^w^
43	Bs-43	27^e^	42^k^	63^h^	68^l^	6.80^l^
44	Bs-44	11^s^	29^t^	36^v^	41.5^D^	4.15^D^
45	Bs-45	6^w^	24^x^	42^s^	48.5^y^	4.85^y^
46	Bs-46	26^f^	52^f^	64^g^	68.5^k^	6.85^k^
47	Bs-47	5^x^	17^B^	28^A^	29.5^M^	2.95^M^
48	Bs-48	17^m^	32^r^	41^t^	44.5^B^	4.45^B^
49	Bs-49	19^k^	31^s^	42^s^	46^z^	4.60^z^
50	Bs-50	36^a^	53^e^	72^c^	80^c^	8.00^c^
51	Bs-51	13^q^	28^u^	36^v^	39^H^	3.90^H^
	C.D.	0.079	0.17	0.213	0.236	
	SE(m)	0.028	0.06	0.076	0.084	
	SE(d)	0.04	0.086	0.107	0.119	
	C.V.	2.772	2.964	2.839	2.712	

*Mean of three replications. Different letters are significantly different at *P* ≤ 0.05 according to Duncan’s Multiple Range Test (DMRT).

**Table 3:** Colony characteristics of *Bipolaris sorokiniana* isolates on Potato Dextrose Agar (PDA).

**S. No.**	**Isolate**	**Colony colour**	**Exudation**	**Zonation**	**Growth pattern**	**Margin**
1	Bs-1	Black	P	P	Smooth appressed	Irregular
2	Bs-2	Whitish	P	A	Smooth appressed	Regular
3	Bs-3	Black	P	A	Smooth appressed	Regular
4	Bs-4	Dark gray	P	P	Rough raised	Irregular
5	Bs-5	Black	A	P	Smooth appressed	Irregular
6	Bs-6	Light gray	P	A	Smooth appressed	Regular
7	Bs-7	Whitish	P	P	Smooth raised	Regular
8	Bs-8	Light gray	P	A	Rough raised	Irregular
9	Bs-9	Whitish	P	A	Rough raised	Regular
10	Bs-10	Dark gray	P	P	Rough raised	Regular
11	Bs-11	Grey	P	A	Smooth appressed	Irregular
12	Bs-12	Dark gray	P	A	Smooth appressed	Regular
13	Bs-13	Light gray	P	P	Smooth appressed	Regular
14	Bs-14	Light gray	A	A	Rough raised	Regular
15	Bs-15	Black	P	A	Smooth appressed	Irregular
16	Bs-16	Light gray	P	A	Smooth appressed	Regular
17	Bs-17	Dark gray	P	P	Smooth appressed	Regular
18	Bs-18	Dark gray	P	P	Rough raised	Regular
19	Bs-19	Light gray	P	A	Smooth appressed	Regular
20	Bs-20	Light gray	P	P	Rough raised	Regular
21	Bs-21	Dark gray	P	A	Smooth appressed	Irregular
22	Bs-22	White	P	P	Smooth appressed	Irregular
23	Bs-23	Light gray	P	P	Rough raised	Irregular
24	Bs-24	Dark gray	P	A	Rough raised	Regular
25	Bs-25	Black	P	P	Rough raised	Regular
26	Bs-26	Grey	P	A	Smooth appressed	Regular
27	Bs-27	Light gray	P	A	Rough raised	Irregular
28	Bs-28	Dark gray	P	A	Rough raised	Irregular
29	Bs-29	Whitish	P	P	Smooth appressed	Regular
30	Bs-30	Black	P	A	Smooth appressed	Regular
31	Bs-31	Black	P	P	Smooth appressed	Regular
32	Bs-32	Black	P	P	Smooth appressed	Regular
33	Bs-33	Whitish	P	P	Rough raised	Regular
34	Bs-34	Light gray	P	P	Smooth appressed	Regular
35	Bs-35	Black	P	P	Rough raised	Regular
36	Bs-36	Whitish	P	A	Smooth appressed	Regular
37	Bs-37	Dark gray	A	A	Smooth appressed	Regular
38	Bs-38	Black	P	A	Smooth raised	Irregular
39	Bs-39	Black	P	P	Rough raised	Irregular
40	Bs-40	Light gray	P	P	Rough raised	Irregular
41	Bs-41	Dark gray	P	P	Smooth appressed	Regular
42	Bs-42	Whitish	P	P	Rough raised	Irregular
43	Bs-43	Whitish	A	A	Smooth appressed	Regular
44	Bs-44	Gray	A	A	Smooth appressed	Irregular
45	Bs-45	Light gray	P	A	Smooth appressed	Regular
46	Bs-46	Light gray	P	P	Smooth appressed	Regular
47	Bs-47	Gray	P	P	Rough raised	Regular
48	Bs-48	Gray	P	P	Rough raised	Irregular
49	Bs-49	Light gray	P	P	Rough raised	Regular
50	Bs-50	White	P	A	Smooth appressed	Regular
51	Bs-51	Gray	P	P	Rough raised	Irregular

Colony color ranged from creamy white to black, with isolates classified into five distinct colour categories: creamy white, light grey, grey, dark grey, and black. Texture differentiated the isolates into smooth and rough types, with 30 isolates forming smooth colonies and 21 forming rough colonies. Most colonies were appressed; however, 23 isolates exhibited raised growth. Colony margins were predominantly regular, although a subset, including Bs-1, Bs-4, Bs-5, Bs-8, Bs-11, Bs-15, Bs-21, Bs-22, Bs-23, Bs-27, Bs-28, Bs-38, Bs-39, Bs-40, Bs-42, Bs-44, Bs-48, and Bs-51, displayed irregular margins. Zonation was present in most isolates but absent in 24 isolates across the color groups. Exudation was common, except in five isolates (Bs-5, Bs-14, Bs-37, Bs-43, and Bs-44), which produced no visible exudates.

### 3.2 Morphological variability

**PLATE 2:**
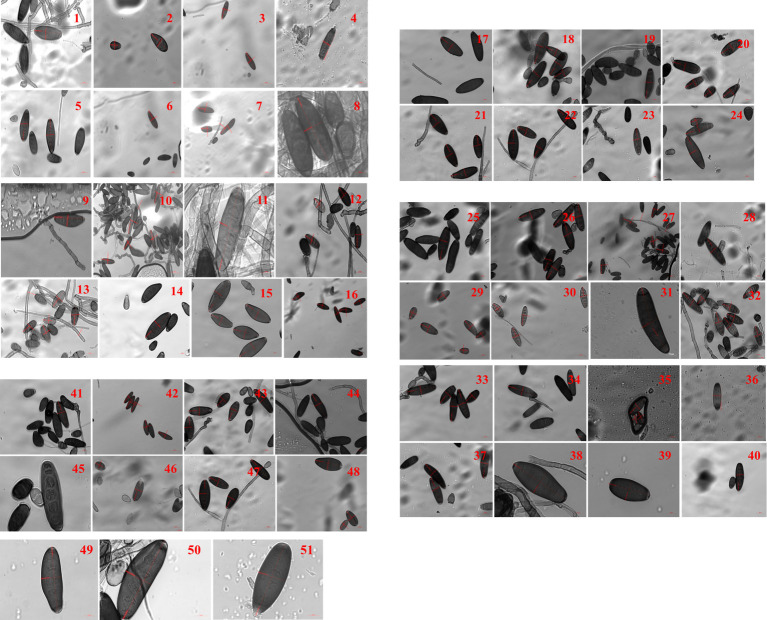
Conidial morphology of *Bipolaris sorokiniana* isolates showing variability in size, shape, and septation patterns. Images 1–51 correspond to isolates Bs-1 to Bs-51, respectively. Scale bars provided with each micrograph.

Significant morphological variation was identified among *B. sorokiniana* isolates based on conidial measurements and septation patterns (Table 4 and Plate 2). Conidial length ranged from 42.37 to 72.02 μm and breadth from 14.61 to 25.18 μm. Isolate Bs-3 produced the largest conidia (72.02 × 25.18 μm), while isolate Bs-12 produced the smallest (42.37 × 14.61 μm). The number of septa ranged from 3 to 11, with mean septation values of 4.2–7.4. Isolates Bs-3, Bs-17, Bs-32, and Bs-44 exhibited the highest septations, whereas Bs-12 and Bs-13 had the lowest number of septa. Conidia were consistently brown to dark brown, with no observed deviations from species-typical pigmentation.

**Table 4:** Morphological characteristics of *Bipolaris sorokiniana* isolates under study.

**S. No.**	**Isolate**	Conidia length (μm)*	Conidia breadth (μm)*	Number of septa*
1	Bs-1	58.23l^m^	22.487^cdefgh^	6.60^abcde^
2	Bs-2	50.42^qrs^	19.014^opqrstuv^	4.80^hijk^
3	Bs-3	72.02^a^	25.176^a^	7.40^a^
4	Bs-4	58.97^jklm^	19.733^klmnopqrstuv^	5.80^cdefghi^
5	Bs-5	61.00^hijk^	21.653^fghijk^	6.40^abcdef^
6	Bs-6	51.49^pqr^	21.706^efghij^	5.20^fghijk^
7	Bs-7	49.41^rs^	18.822^qrstuv^	4.60^ijk^
8	Bs-8	51.7^pqr^	19.224^nopqrstuv^	5.40^efghijk^
9	Bs-9	58.86^klm^	21.306^ghijklm^	6.60^abcde^
10	Bs-10	51.99^pqr^	21.658^fghijk^	6.20^abcdefg^
11	Bs-11	51.13^pqrs^	16.053^w^	4.60^ijk^
12	Bs-12	42.37^u^	14.606^w^	4.20^k^
13	Bs-13	46.40^t^	18.426^stuv^	4.20^k^
14	Bs-14	58.89^klm^	24.865^ab^	5.40^efghijk^
15	Bs-15	63.62^efg^	24.952^ab^	6.20^abcdefg^
16	Bs-16	63.91^def^	23.569^abcde^	7.20^ab^
17	Bs-17	68.67^b^	24.276^abc^	7.40^a^
18	Bs-18	51.26^pqrs^	21.821^efghi^	5.40^efghijk^
19	Bs-19	50.31^qrs^	20.855^ghijklmnop^	5.20^fghijk^
20	Bs-20	48.83^s^	19.41^mnopqrstuv^	4.80^hijk^
21	Bs-21	61.8^fghi^	23.883^abc^	6.20^abcdefg^
22	Bs-22	57.86^lm^	21.015^ghijklmn^	5.80^cdefghi^
23	Bs-23	44.15^tu^	18.266^tuv^	5.20^fghijk^
24	Bs-24	71.33^a^	23.329^bcdef^	7.00^abc^
25	Bs-25	66.22^cd^	23.307^bcdef^	6.80^abcd^
26	Bs-26	50.46^qrs^	19.646^lmnopqrstuv^	5.60^defghij^
27	Bs-27	57.88^lm^	20.836^ghijklmnop^	6.80^abcd^
28	Bs-28	51.37^pqrs^	18.546^rstuv^	7.00^abc^
29	Bs-29	61.33^ghij^	15.816^w^	5.40^efghijk^
30	Bs-30	64.02^def^	21.062^ghijklmn^	6.20^abcdefg^
31	Bs-31	59.41^ijkl^	19.686^lmnopqrstuv^	6.00^bcdefgh^
32	Bs-32	64.14^def^	21.991^defghi^	7.40^a^
33	Bs-33	50.06^qrs^	19.851^jklmnopqrstu^	5.80^cdefghi^
34	Bs-34	45.83^t^	19.801^jklmnopqrstuv^	7.00^abc^
35	Bs-35	43.49^u^	15.768^w^	4.40^jk^
36	Bs-36	44.95^tu^	18.075^uv^	4.60^ijk^
37	Bs-37	53.42^op^	17.912^v^	5.60^defghij^
38	Bs-38	58.19^lm^	22.432^cdefgh^	6.40^abcdef^
39	Bs-39	51.92^pqr^	18.935^pqrstuv^	4.80^hijk^
40	Bs-40	60.78^hijk^	21.038^ghijklmn^	5.40^efghijk^
41	Bs-41	64.24^def^	21.679^fghijk^	6.00^bcdefgh^
42	Bs-42	64.19^def^	22.592^cdefg^	6.20^abcdefg^
43	Bs-43	53.31^op^	20.168^ijklmnopqrst^	5.60^defghij^
44	Bs-44	67.19^bc^	21.498^fghijkl^	7.40^a^
45	Bs-45	54.93^no^	20.919^ghijklmno^	5.60^defghij^
46	Bs-46	62.04^fgh^	20.261^ijklmnopqrs^	6.80^abcd^
47	Bs-47	65.98^cde^	23.80^abcd^	6.60^abcde^
48	Bs-48	52.34^pq^	21.121^ghijklmn^	5.80^cdefghi^
49	Bs-49	50.63^qrs^	20.596^hijklmnopq^	5.00^ghijk^
50	Bs-50	56.66^mn^	21.505^fghijkl^	5.60^defghij^
51	Bs-51	56.9^lmn^	20.360^ijklmnopqr^	6.00^bcdefgh^
	C.D.	2.14	1.591	0.998
	SE(m)	0.769	0.566	0.358
	SE(d)	1.087	0.801	0.506

*Mean of five replications. Different letters are significantly different at *P* ≤ 0.05 according to Duncan’s Multiple Range Test (DMRT).

**Figure 3:**
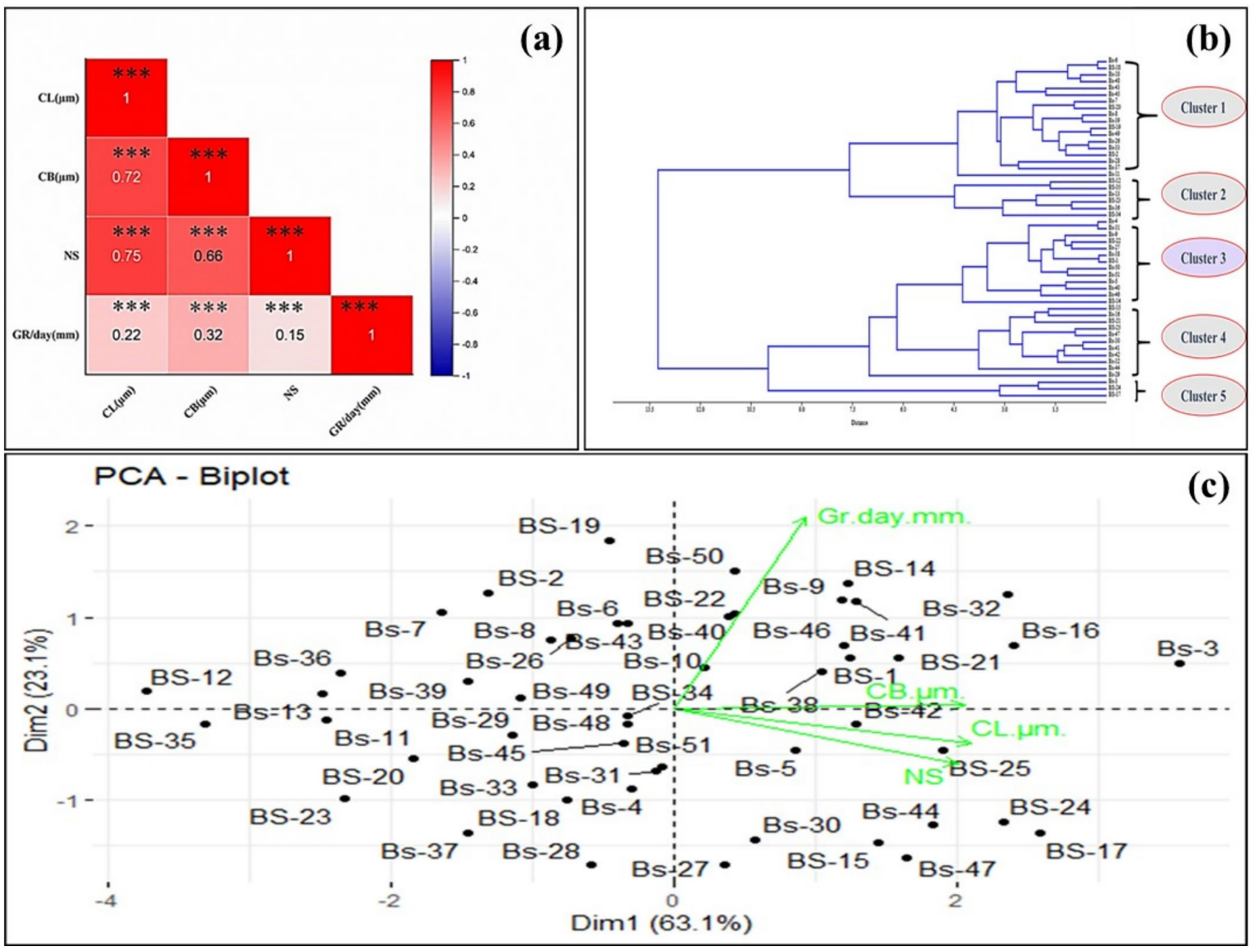
**(a)** Correlogram showing the correlation matrix among the morpho-cultural traits evaluated across *Bipolaris sorokiniana* isolates. *** indicates significance at the 0.01% level. **(b)** Hierarchical clustering of *B. sorokiniana* isolates based on the assessed morpho-cultural characteristics. **(c)** Principal component analysis (PCA) depicting the grouping patterns and trait contributions among the isolates.

Correlation analysis demonstrated strong positive associations among conidial length, conidial breadth, and conidial septation (Figure 3a). Conidial length was positively correlated with breadth (*r* = 0.72) and septation (*r* = 0.75), while breadth was positively correlated with septation (*r* = 0.66). Growth rate exhibited weaker positive associations with these traits. No significant correlation was observed between growth rate and other cultural characteristics.

Cluster analysis of quantitative morpho-cultural variables grouped the isolates into five distinct clusters (Figure 3b). Cluster 1 included isolates with the smallest conidia and lowest septation, whereas Cluster 5 comprised isolates with the largest conidia, highest septation, and the highest growth rates. The remaining clusters represented intermediate morpho types. Principal component analysis corroborated these groupings (Figure 3c). The first principal component (PC1) accounted for 63.1% of the total variation and was primarily influenced by conidial length, breadth, and septation. The second principal component (PC2) explained 23.1% of the variation and was mainly associated with growth rate. Together, PC1 and PC2 explained 86.2% of the overall variation, and the PCA ordination revealed clear separation of clusters along these axes.

### 3.3 Pathogenic variability

Substantial pathogenic variation was detected among *B. sorokiniana* isolates for all disease-associated traits evaluated on the susceptible cultivar Sonalika and the resistant cultivar Chirya-3 (Tables 5, 6). The incubation period, lesion morphology, infection frequency, lesion coverage, disease severity, and percent disease index each displayed wide quantitative ranges, reflecting considerable phenotypic heterogeneity. In Sonalika, the incubation period ranged from 2.75 to 6.00 days, while in Chirya-3 it ranged from 3.25 to 6.75 days. Isolates Bs-3, Bs-15, and Bs-5 exhibited the shortest incubation periods. Lesion dimensions were also highly variable in Sonalika, lesion length ranged from 1.95 to 5.05 mm and lesion size from 1.81 to 8.53 mm^2^, with Bs-3 consistently producing the largest lesions. In contrast, Chirya-3 showed reduced lesion expansion, with lesion length from 1.23 to 2.40 mm and lesion size from 1.16 to 2.88 mm^2^, indicative of partial resistance. Infection frequency varied markedly among isolates (Sonalika: 4.0 to 8.5 lesions/cm^2^; Chirya-3: 1.25 to 3.75 lesions/cm^2^), and lesion coverage ranged from 2.5 to 67.5% in Sonalika and 2.5 to 22.5% in Chirya-3. Isolates Bs-3, Bs-21, and Bs-24 produced the highest lesion coverage values. Disease severity and percent disease index exhibited similar trends: in Sonalika, disease severity ranged from 1.0 to 4.5 and percent disease index from 8.75 to 90.0, whereas in Chirya-3, disease severity ranged from 0.25 to 2.25 and percent disease index from 1.0 to 20.0. Across all traits and hosts, Bs-3 consistently ranked among the most aggressive isolates, displaying the shortest incubation period, largest lesions, highest infection frequency, maximum lesion coverage, and the greatest disease severity and percent disease index.

**Table 5:** Pathogenicity attributes of *Bipolaris sorokiniana* isolates on the wheat cultivar Sonalika.

Isolate	IP (days)*	LL (mm)*	LB (mm)*	LS (mm^2^)	IF (lesions/cm^2^)	LC (%)	DS	PDI
Bs-1	4.25^cdefgh^	3.78^e^	1.25^cdefgh^	4.72^ghijk^	6.25^efghij^	24.38	3.25^bcdef^	65.00
Bs-2	5.00^abcde^	2.50^klm^	1.10^fghij^	2.75^mnop^	6.50^defghi^	10.00	2.00^ghij^	40.00
Bs-3	2.75^i^	5.05^a^	1.69^a^	8.53^a^	8.25^ab^	67.50	4.50^a^	90.00
Bs-4	4.50^bcdefg^	3.45^efgh^	1.33^bcdefgh^	4.58^ghijk^	6.50^defghi^	12.50	2.50^efghi^	50.00
Bs-5	3.25^ghi^	4.83^ab^	1.47^abcde^	7.10^bc^	6.75^cdefgh^	63.75	4.25^ab^	85.00
Bs-6	5.00^abcde^	3.50^efgh^	1.20^defghi^	4.20^hijkl^	5.75^ghijkl^	22.50	3.00^cdefg^	60.00
Bs-7	4.25^cdefgh^	3.68^ef^	0.93^ij^	3.42^klmn^	5.75^ghijkl^	22.50	3.00^cdefg^	60.00
Bs-8	4.00^defghi^	3.55^efgh^	1.33^bcdefgh^	4.72^ghijk^	6.25^efghij^	35.00	3.50^abcde^	70.00
Bs-9	4.50^bcdefg^	4.65^abc^	1.37^abcdefg^	6.37^bcde^	6.75^cdefgh^	37.50	3.75^abcd^	75.00
Bs-10	4.00^defghi^	3.65^efg^	1.43^abcde^	5.21^efghij^	7.00^bcdefg^	35.00	3.50^abcde^	70.00
Bs-11	4.25^cdefgh^	1.95^n^	0.93^ij^	1.81^p^	4.75^klmn^	22.50	3.00^cdefg^	60.00
Bs-12	5.25^abcd^	2.10^mn^	0.87^j^	1.82^p^	4.00^n^	8.75	1.75^hij^	35.00
Bs-13	5.50^abc^	1.98^n^	1.07^ghij^	2.11^op^	4.25^mn^	2.50	1.00^j^	20.00
Bs-14	5.25^abcd^	3.10^hij^	1.53^abc^	4.74^ghijk^	6.50^defghi^	13.75	2.75^defgh^	55.00
Bs-15	2.75^i^	4.91^a^	1.47^abcde^	7.21^abc^	7.00^bcdefg^	50.00	4.00^abc^	80.00
Bs-16	3.25^ghi^	4.78^ab^	1.40^abcdef^	6.69^bc^	7.50^abcde^	63.75	4.25^ab^	85.00
Bs-17	3.25^ghi^	4.81^ab^	1.20^defghi^	5.77^bcd^	7.25^abcdef^	37.50	3.75^abcd^	75.00
Bs-18	3.50^fghi^	3.23^fghi^	1.53^abc^	4.94^ghijk^	6.00^fghijk^	35.00	3.50^abcde^	70.00
Bs-19	5.25^abcd^	2.68^jkl^	1.17^efghij^	3.13^lmnop^	4.75^klmn^	18.75	2.50^efghi^	50.00
Bs-20	5.25^abcd^	2.23^lmn^	0.93^ij^	2.07^op^	5.00^jklmn^	7.50	1.50^ij^	30.00
Bs-21	3.25^ghi^	4.73^ab^	1.50^abcd^	7.09^abc^	8.00^abc^	67.50	4.50^a^	90.00
Bs-22	3.25^ghi^	3.78^e^	1.40^abcdef^	5.29^efghi^	6.25^efghij^	50.00	4.00^abc^	80.00
Bs-23	5.75^ab^	2.25^lmn^	1.03^hij^	2.31^nop^	4.75^klmn^	7.50	1.50^ij^	30.00
Bs-24	3.00^hi^	4.97^a^	1.50^abcd^	7.45^abc^	8.50^a^	67.50	4.50^a^	90.00
Bs-25	3.50^fghi^	4.87^a^	1.63^ab^	7.93^ab^	7.50^abcde^	50.00	4.00^abc^	80.00
Bs-26	5.75^ab^	3.33^efgh^	1.33^bcdefgh^	4.42^ghijk^	5.25^ijklmn^	16.88	2.25^fghi^	45.00
Bs-27	5.50^abc^	3.48^efgh^	1.40^abcdef^	4.87^ghijk^	6.50^defghi^	24.38	3.25^bcdef^	65.00
Bs-28	3.50^fghi^	4.83^ab^	1.27^cdefgh^	6.13^bc^	6.75^cdefgh^	40.00	4.00^abc^	80.00
Bs-29	6.00^a^	3.20^fghi^	1.33^bcdefgh^	4.25^hijkl^	5.25^ijklmn^	16.88	2.25^fghi^	45.00
Bs-30	3.25^ghi^	4.75^ab^	1.37^abcdefg^	6.50^bcd^	7.00^bcdefg^	63.75	4.25^ab^	85.00
Bs-31	5.00^abcde^	3.65^efg^	1.43^abcde^	5.21^efghij^	6.00^fghijk^	32.50	3.25^bcdef^	65.00
Bs-32	3.25^ghi^	4.63^abc^	1.27^cdefgh^	5.88^cdef^	7.00^bcdefg^	53.13	4.25^ab^	85.00
Bs-33	3.75^efghi^	3.48^efgh^	1.47^abcde^	5.11^fghij^	5.75^ghijkl^	32.50	3.25^bcdef^	32.50
Bs-34	4.00^defghi^	3.60^efgh^	1.30^cdefgh^	4.68^ghijk^	5.75^ghijkl^	18.75	2.50^efghi^	18.75
Bs-35	5.00^abcde^	3.15^ghi^	1.17^efghij^	3.68^klm^	5.25^ijklmn^	10.00	2.00^ghij^	10.00
Bs-36	5.50^abc^	2.48^klm^	0.93^ij^	2.30^nop^	4.50^lmn^	8.75	1.75^hij^	8.75
Bs-37	4.75^abcdef^	3.35^efgh^	1.27^cdefgh^	4.25^ijkl^	5.75^ghijkl^	20.63	2.75^defgh^	20.63
Bs-38	3.50^fghi^	3.33^efgh^	1.38^abcdefg^	4.59^ghijk^	6.00^fghijk^	32.50	3.25^bcdef^	32.50
Bs-39	5.25^abcd^	3.35^efgh^	1.33^bcdefgh^	4.45^ghijk^	5.75^ghijkl^	16.88	2.25^fghi^	16.88
Bs-40	4.00^defghi^	3.43^efgh^	1.20^defghi^	4.11 s^hijkl^	5.50^hijklm^	32.50	3.25^bcdef^	32.50
Bs-41	3.75^efghi^	4.35^bc^	1.27^cdefgh^	5.52^defgh^	6.25^efghij^	63.75	4.25^ab^	63.75
Bs-42	3.75^efghi^	4.23^cd^	1.37^abcdefg^	5.79^defg^	6.50^defghi^	50.00	4.00^abc^	50.00
Bs-43	4.75^abcdef^	3.40^efgh^	1.10^fghij^	3.74^jklm^	5.25^ijklmn^	20.63	2.75^defgh^	20.63
Bs-44	3.50^fghi^	4.83^ab^	1.53^abc^	7.38^ab^	7.75^abcd^	63.75	4.25^ab^	63.75
Bs-45	3.75^efghi^	3.53^efgh^	1.30^cdefgh^	4.58^ghijk^	5.75^ghijkl^	35.00	3.5^abcde^	35.00
Bs-46	3.75^efghi^	4.70^ab^	1.47^abcde^	6.90^bc^	8.00^abc^	40.00	4.00^abc^	40.00
Bs-47	3.00^hi^	4.88^a^	1.57^abc^	7.66^abc^	7.75^abcd^	40.00	4.00^abc^	40.00
Bs-48	4.00^defghi^	3.83^de^	1.40^abcdef^	5.36^defg^	6.25^efghij^	32.50	3.25^bcdef^	32.50
Bs-49	5.25^abcd^	2.83^ijk^	1.07^ghij^	3.02^lmno^	4.00^n^	18.75	2.50^efghi^	18.75
Bs-50	3.75^efghi^	3.48^efgh^	1.43^abcde^	4.97^ghijk^	6.25^efghij^	32.50	3.25^bcdef^	32.50
Bs-51	4.00^defghi^	3.35^efgh^	1.20^defghi^	4.02^ijkl^	6.50^defghi^	35.00	3.50^abcde^	35.00
C.D.	0.972	1.042	0.412	0.259	1.06	1.175		
SE(m)	0.348	0.373	0.147	0.093	0.379	0.42		
SE(d)	0.492	0.527	0.208	0.131	0.536	0.594		
C.V.	21.693	17.681	7.979	14.281	15.405	13.56		

*Mean of four replications. Different letters are significantly different at *P* ≤ 0.05 according to Duncan’s Multiple Range Test (DMRT). IP-Incubation Period, LL- Lesion length, LB-Lesion breadth, LS-Lesion Size, IF-Infection Frequency, LC-Lesion Cover, DS-Disease severity, PDI-Percent disease index.

**Table 6:** Pathogenicity attributes of *Bipolaris sorokiniana* isolates on the wheat cultivar Chirya-3.

Isolate	IP (days)*	LL (mm)*	LB (mm)*	LS (mm^2^)	IF (lesions/cm^2^)	LC (%)	DS	PDI
Bs-1	6.00^abcd^	2.05^cdefghi^	1.08^abcdefgh^	2.21^defghijklmn^	2.50^bcdef^	10.00	1.25^bcde^	8.00
Bs-2	6.50^ab^	1.88^hijk^	0.93^gh^	1.74^nopqrs^	1.75^efg^	12.50	1.50^abcd^	9.00
Bs-3	3.75^ghi^	2.35^ab^	1.20^abc^	2.82^a^	3.25^abc^	22.50	2.25^a^	17.00
Bs-4	5.50^abcdef^	2.00^cdefghij^	1.05^abcdefgh^	2.10^fghijklmno^	2.50^bcdef^	7.50	0.75^def^	7.00
Bs-5	3.25^i^	2.10^cdefgh^	1.15^abcde^	2.41^abcdefghijk^	2.75^abcde^	10.00	1.00^cdef^	8.00
Bs-6	6.25^abc^	2.00^cdefghij^	1.05^abcdefgh^	2.10^fghijklmno^	2.50^bcdef^	17.50	1.75^abc^	7.00
Bs-7	6.75^a^	1.80^ijklm^	0.95^fgh^	1.71^nopqrst^	2.00^defg^	10.00	1.00^cdef^	8.00
Bs-8	6.50^ab^	1.90^ghij^	1.10^abcdefg^	2.09^fghijklmno^	2.25^cdefg^	12.50	1.25^bcde^	9.00
Bs-9	4.50^defghi^	2.15^bcdefg^	1.18^abcd^	2.53^abcdefg^	2.75^abcde^	12.50	1.25^bcde^	9.00
Bs-10	4.75^cdefghi^	1.95^efghij^	1.00^bdefgh^	1.95^jklmnopq^	2.25^cdefg^	12.50	1.25^bcde^	9.00
Bs-11	6.75^a^	1.25^p^	0.95^fgh^	1.18^u^	1.50^fg^	10.00	1.00^cdef^	8.00
Bs-12	6.50^ab^	1.33^op^	0.95^fgh^	1.26^tu^	1.50^fg^	5.00	0.50^ef^	6.00
Bs-13	6.75^a^	1.23^p^	0.95^fgh^	1.16^u^	1.25^g^	7.50	0.75^def^	3.00
Bs-14	5.50^abcdef^	2.03^cdefghi^	1.00^bdefgh^	2.03^hijklmno^	2.50^bcdef^	10.00	1.00^cdef^	8.00
Bs-15	4.25^efghi^	2.08^cdefgh^	1.18^abcd^	2.45^abcdefghij^	3.00^abcd^	12.50	1.25^bcde^	5.00
Bs-16	4.00^fghi^	2.15^bcdefg^	1.20^ab^	2.58^abcdef^	3.00^abcd^	15.00	1.50^abcd^	10.00
Bs-17	3.75^ghi^	2.05^cdefghi^	1.13^abcdef^	2.31^bcdefghijkl^	2.75^abcde^	7.50	0.75^def^	7.00
Bs-18	5.75^abcde^	1.98^defghij^	1.05^abcdefgh^	2.07^ghijklmno^	2.25^cdefg^	10.00	1.00^cdef^	8.00
Bs-19	6.00^abcd^	1.53^no^	0.98^efgh^	1.49^qrstu^	1.50^fg^	7.50	0.75^def^	7.00
Bs-20	6.50^ab^	1.50^no^	0.95^fgh^	1.42^rstu^	1.75^efg^	7.50	0.75^def^	3.00
Bs-21	3.75^ghi^	2.40^a^	1.20^ab^	2.88^a^	3.50^ab^	17.50	1.75^abc^	15.00
Bs-22	5.75^abcde^	2.03^cdefghi^	1.00^bdefgh^	2.03^hijklmno^	2.75^abcde^	7.50	0.75^def^	7.00
Bs-23	6.75^a^	1.45^nop^	0.90^h^	1.30^stu^	1.50^fg^	10.00	1.00^cdef^	8.00
Bs-24	4.00^fghi^	2.25^abc^	1.18^abcd^	2.65^abcd^	3.50^ab^	20.00	2.00^ab^	20.00
Bs-25	3.50^hi^	2.23^abcd^	1.23^a^	2.74^ab^	3.75^a^	20.00	2.00^ab^	16.00
Bs-26	6.25^abc^	1.88^hijkl^	1.00^bdefgh^	1.88^lmnopqr^	2.00^defg^	10.00	1.00^cdef^	8.00
Bs-27	5.50^abcdef^	2.05^cdefghi^	1.08^abcdefgh^	2.21^defghijklmn^	2.25^cdefg^	12.50	1.25^bcde^	9.00
Bs-28	4.25^efghi^	2.10^cdefgh^	1.08^abcdefgh^	2.26^bcdefghijklm^	2.75^abcde^	10.00	1.00^cdef^	8.00
Bs-29	6.00^abcd^	1.95^efghij^	0.95^fgh^	1.85^lmnopqr^	2.25^cdefg^	5.00	0.50^ef^	6.00
Bs-30	3.50^hi^	2.13^bcdefgh^	1.05^abcdefgh^	2.23^cdefghijklm^	2.75^abcde^	10.00	1.00^cdef^	8.00
Bs-31	5.25^abcdefg^	1.98^defghij^	1.08^abcdefgh^	2.13^efghijklmno^	2.75^abcde^	7.50	0.75^def^	7.00
Bs-32	4.50^defghi^	2.05^cdefghi^	1.13^abcdef^	2.31^bcdefghijkl^	2.75^abcde^	12.50	1.25^bcde^	5.00
Bs-33	5.50^abcdef^	2.00^cdefghij^	1.15^abcde^	2.30^bcdefghijkl^	2.00^defg^	10.00	1.00^cdef^	8.00
Bs-34	6.50^ab^	2.03^cdefghi^	1.03^bcdefgh^	2.09^ghijklmno^	2.25^cdefg^	12.50	1.25^bcde^	5.00
Bs-35	6.75^a^	1.65^klmn^	1.00^bdefgh^	1.65^opqrstu^	1.25^g^	5.00	0.50^ef^	6.00
Bs-36	6.25^abc^	1.76^jklm^	0.88^h^	1.55^pqrstu^	1.50^fg^	2.50	0.25^f^	1.00
Bs-37	6.00^abcd^	2.05^cdefghi^	0.95^fgh^	1.94^jklmnopq^	1.75^efg^	7.50	0.75^def^	3.00
Bs-38	5.00^bcdefgh^	1.93^fghij^	1.00^bdefgh^	1.93^klmnopq^	2.25^cdefg^	15.00	1.50^abcd^	6.00
Bs-39	6.00^abcd^	2.18^abcdef^	1.03^bcdefgh^	2.24^cdefghijklm^	1.75^efg^	7.50	0.75^def^	7.00
Bs-40	5.00^bcdefgh^	1.93^fghij^	1.00^bdefgh^	1.93^klmnopq^	2.25^cdefg^	10.00	1.00^cdef^	4.00
Bs-41	4.25^efghi^	2.20^abcde^	1.15^abcde^	2.53^abcdefg^	2.50^bcdef^	10.00	1.00^cdef^	8.00
Bs-42	3.75^ghi^	2.18^abcdef^	1.13^abcdef^	2.46^abcdefghi^	2.50^bcdef^	7.50	0.75^def^	7.00
Bs-43	6.75^a^	2.00^cdefghij^	1.00^bdefgh^	2.00^ijklmnop^	2.25^cdefg^	7.50	0.75^def^	3.00
Bs-44	4.00^fghi^	2.25^abc^	1.20^abc^	2.70^abc^	3.25^abc^	17.50	1.75^abc^	15.00
Bs-45	5.50^abcdef^	2.00^cdefghij^	1.05^abcdefgh^	2.10^fghijklmno^	2.00^defg^	7.50	0.75^def^	7.00
Bs-46	3.75^ghi^	2.13^bcdefgh^	1.18^abcd^	2.51^abcdefgh^	3.25^abc^	17.50	1.75^abc^	19.00
Bs-47	4.25^efghi^	2.23^abcd^	1.18^abcd^	2.63^abcde^	3.50^ab^	15.00	1.50^abcd^	14.00
Bs-48	5.25^abcdefg^	2.20^abcde^	1.13^abcdef^	2.48^abcdefghi^	2.00^defg^	7.50	0.75^def^	7.00
Bs-49	6.25^abc^	1.80^ijklm^	1.00^bdefgh^	1.80^mnopqr^	1.50^fg^	15.00	1.50^abcd^	6.00
Bs-50	5.75^abcde^	2.03^cdefghi^	1.15^abcde^	2.33^bcdefghijkl^	2.50^bcdef^	10.00	1.00^cdef^	4.00
Bs-51	5.50^abcdef^	2.15^bcdefg^	1.18^abcd^	2.53^abcdefg^	2.25^cdefg^	12.50	1.25^bcde^	5.00
CD	0.688	1.251	0.2	0.16	0.397	0.966		
SE(m)	0.246	0.447	0.072	0.057	0.142	0.345		
SE(d)	0.348	0.633	0.101	0.081	0.201	0.488		
**C. V.**	44.389	16.87	7.272	10.785	13.456	29.235		

*Mean of four replications. Different letters are significantly different at *P* ≤ 0.05 according to Duncan’s Multiple Range Test (DMRT). IP-Incubation Period, LL- Lesion length, LB-Lesion breadth, LS-Lesion Size, IF-Infection Frequency, LC-Lesion Cover, DS-Disease severity, PDI-Percent disease index.

**Figure 4:**
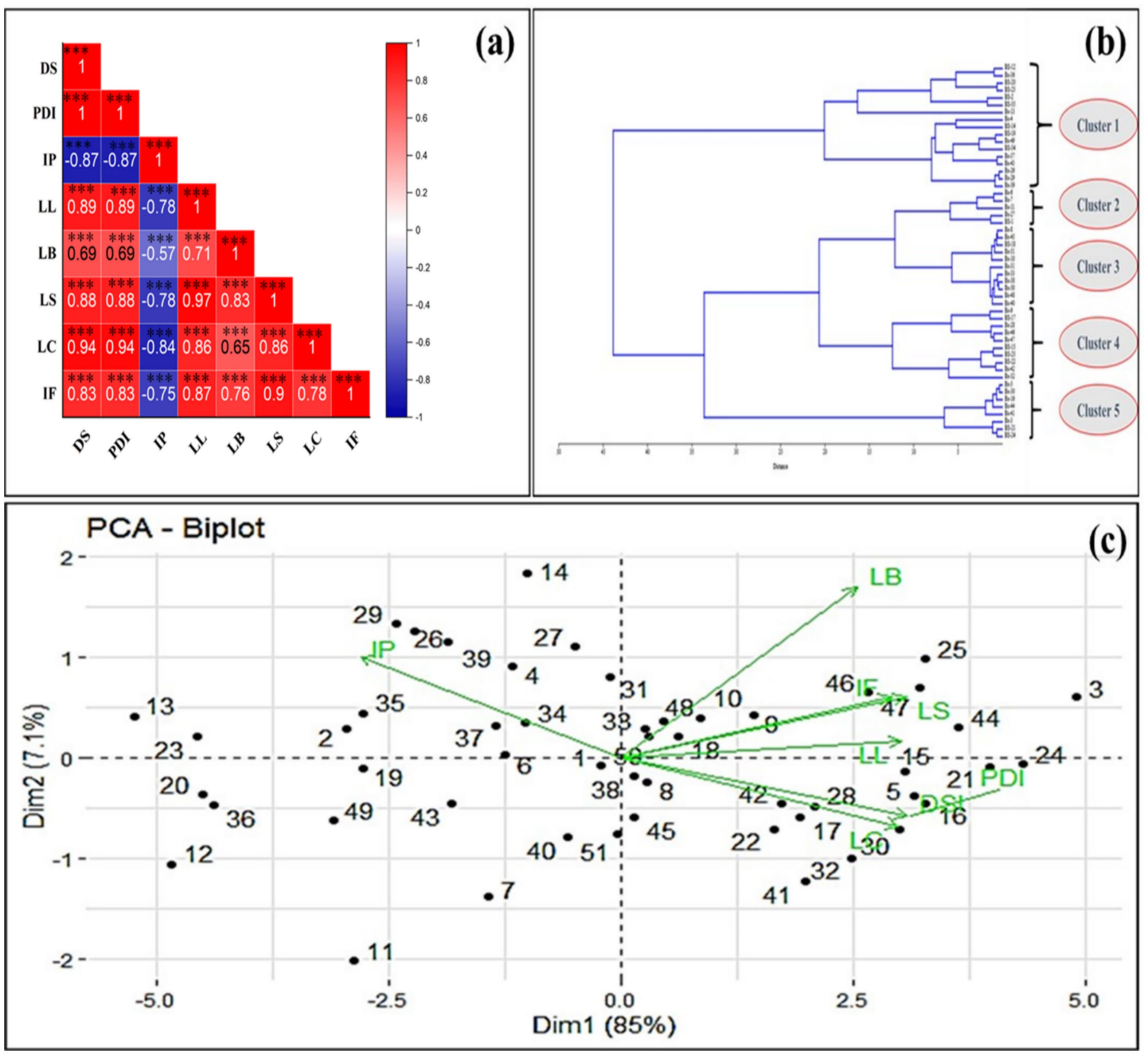
**(a)** Correlogram illustrating the correlation matrix among pathogenicity attributes of *Bipolaris sorokiniana* isolates; *** denotes significance at the 0.01% level. **(b)** Hierarchical clustering of *B. sorokiniana* isolates based on pathogenicity attributes. **(c)** Principal component analysis (PCA) depicting the distribution of isolates and the contribution of pathogenicity attributes to observed variation.

Correlation analysis (Figure 4a) identified a strong negative association between incubation period and disease severity (*r* = −0.87). The incubation period was also negatively correlated with lesion length, lesion breadth, lesion size, lesion coverage, and infection frequency. Conversely, disease severity exhibited highly significant positive correlations with all lesion-related traits, suggesting that virulence is influenced by rapid infection onset, lesion expansion, and tissue colonization. Hierarchical clustering divided isolates into five pathogenicity clusters (Figure 4b). Cluster 1 included isolates with extended incubation periods and minimal lesion development, representing the least virulent group. In contrast, Cluster 5 (comprising Bs-3, Bs-5, Bs-16, Bs-21, Bs-24, Bs-30, Bs-41, and Bs-44) was characterized by the shortest incubation periods and the highest lesion metrics, forming the most virulent assemblage. Principal component analysis supported this grouping, with PC1 (85.0%) primarily reflecting lesion size, lesion breadth, infection frequency, and PC2 (7.1%) associated mainly with lesion coverage and disease severity (Figure 4c). Together, the first two components accounted for 92.1% of the total variation, indicating strong covariation among pathogenicity attributes.

Factorial analysis of variance (ANOVA) revealed highly significant effects (*p* < 0.001) of cultivar., isolate, and genetic subpopulation (SP1 and SP2) on disease severity and percent disease index (PDI) (Table 7). The results indicated substantial variability in pathogenic potential among the tested isolates. The main effect of cultivar was highly significant, with the susceptible cultivar ‘Sonalika’ exhibiting significantly higher disease severity and PDI compared to the resistant cultivar ‘Chirya-3’. Similarly, isolates differed significantly in their virulence, indicating a broad spectrum of aggressiveness within the pathogen population. Subpopulation also had a significant effect on disease expression, where isolates belonging to SP2 showed significantly higher disease severity and PDI than those in SP1, suggesting structured variation in pathogenicity.

**Table 7:** ANOVA for disease severity.

**Source of variation**	**df**	**SS**	**MS**	***F*-value**	***p*-value**
Cultivar (C)	1	185.42	185.42	152.36	<0.001***
Isolates (I)	50	620.75	12.41	10.21	<0.001***
Subpopulation (SP)	1	42.88	42.88	35.24	<0.001***
C × I	50	210.65	4.21	3.46	<0.001***
C × SP	1	18.12	18.12	14.89	<0.001***
I × SP	50	130.45	2.61	2.15	<0.01**
C × I × SP	50	65.32	1.31	1.08	ns
Error	204	248.60	1.22		

Df- Degrees of freedom, SS- Sum of squares, MS- Mean of squares.

**P ≤ 0.01; ***P ≤ 0.001.

Significant interaction effects between cultivar × isolate and isolate × subpopulation (*p* < 0.05) further indicated that the response of wheat cultivars varied depending on the specific isolate and its genetic background. However, the three-way interaction (cultivar × isolate × subpopulation) was not significant, suggesting that the combined effect of these factors did not vary consistently across all conditions. Least square means (LS means) analysis confirmed that ‘Sonalika’ recorded the highest mean disease severity (3.82 ± 0.11) and PDI (71.6 ± 2.1), whereas ‘Chirya-3’ showed significantly lower values (1.48 ± 0.07 and 16.2 ± 1.3, respectively). Among subpopulations, SP2 exhibited significantly higher mean disease severity (3.12 ± 0.10) and PDI (56.3 ± 2.2) compared to SP1 (2.58 ± 0.09 and 44.8 ± 1.8, respectively) (Table 8). Overall, these results demonstrate that both host genotype and pathogen population structure play critical roles in determining disease severity, with isolate-specific effects contributing significantly to the observed variation.

**Table 8:** LS means for Disease severity and PDI.

**Factor**	**Level**	**Severity (Mean ± SE)**	**PDI (Mean ± SE)**
Cultivar	Sonalika	3.82 ± 0.11^a^	71.6 ± 2.1^a^
	Chirya-3	1.48 ± 0.07^b^	16.2 ± 1.3^b^
Subpopulation	SP1	2.58 ± 0.09^b^	44.8 ± 1.8^b^
	SP2	3.12 ± 0.10^a^	56.3 ± 2.2^a^

Different letters are significantly different at *P* ≤ 0.05 according to Duncan’s Multiple Range Test (DMRT).

### 3.4 Molecular variability

Forty-eight *B. sorokiniana* isolates with high-quality genomic DNA were genotyped at 28 polymorphic SSR loci to evaluate molecular variability (Table 1). The SSR primers, which ranged from 18 to 26 base pairs in length and melting temperatures between 59 °C and 66 °C, consistently generated clear amplicons across all isolates. Most loci amplified optimally at 59–60 °C. Alleles were scored based on fragment-size homology, resulting in the identification of 43 alleles in total, with each locus exhibiting one or two alleles. Among the 28 markers, 15 were biallelic, and 13 were monomorphic, producing amplicons between 125 and 265 base pairs. Genetic diversity values ranged from 0.0408 (e.g., BS-28_1, BS-30_1, BS-35_1, BS-39_1, BS070) to 0.4991 (BS-24, BS-28), with a mean of 0.2984. Polymorphism information content (PIC) values varied from 0.0400 to 0.3746 (mean = 0.2406), indicating moderate insightfulness of the SSR panel. Highest genetic diversity demonstrated by markers such as BS-24, BS-28, BS-11, and BS-39 and PIC values, and are therefore informative for assessing population-level variation.

#### 3.4.1 Population structure analysis

**Figure 5:**
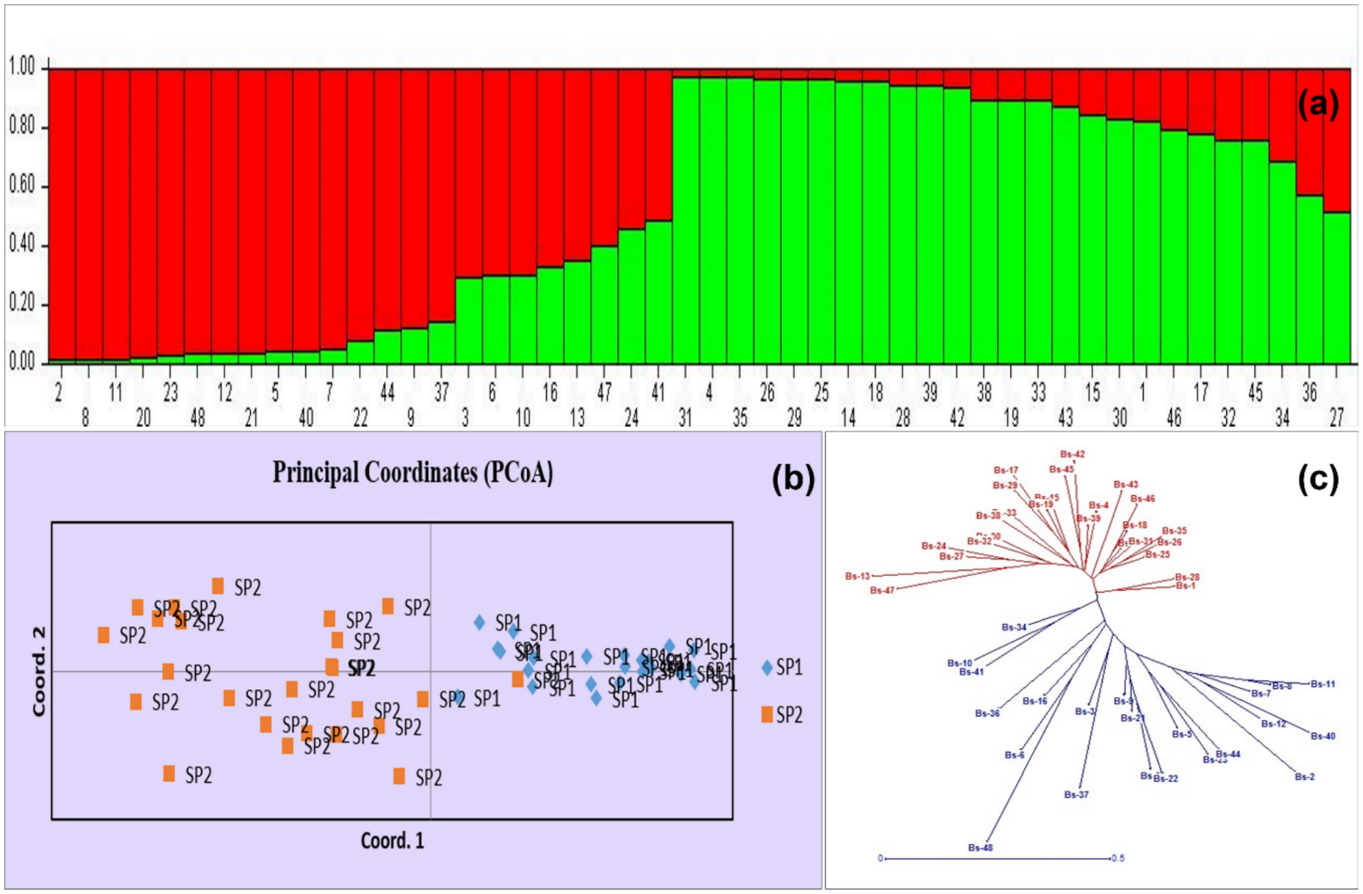
**(a)** Structure bar plot illustrating the inferred population structure of *Bipolaris sorokiniana* isolates. **(b)** Principal coordinates analysis (PCoA) of 48 isolates based on allelic variation across 28 SSR loci. **(c)** Weighted Neighbour-Joining radial tree generated in DARwin 6 showing the genetic relationships among the 48 isolates.

Binary SSR data (one = present, zero = absent) from 28 loci were analyzed in STRUCTURE v2.3.4 using the admixture model with correlated allele frequencies. Each analysis included a 100,000-iteration burn-in followed by 100,000 MCMC iterations, examining K values from 1 to 10 with five replicates per K. ΔK values calculated in STRUCTURE Harvester exhibited a pronounced peak at K = 2 (ΔK = 72.38), indicating that the 48 *B. sorokiniana* isolates were divided into two distinct genetic clusters (Table 9). The STRUCTURE bar plot at K = 2 separated the isolates into two subpopulations: 23 isolates were primarily assigned to subpopulation 1 (red), and 25 isolates to subpopulation 2 (green) (Figure 5A and Table 10). Membership coefficient (Q-value) patterns demonstrated predominantly discrete groupings, with a small number of admixed isolates, indicating limited but detectable gene flow.

**Table 9:** Evanno table generated by STRUCTURE Harvester.

**K**	**Reps**	**Delta K**
1	5	NA
2	5	72.3826
3	5	0.38338
4	5	0.07873
5	5	0.78963
6	5	0.18888
7	5	2.13534
8	5	0.30759
9	5	1.64402
10	5	NA

**Table 10:** Subpopulation assignment and isolate grouping based on STRUCTURE analysis.

**Subpopulation**	**Number**	**Isolates**
SP1	23	Bs-2, Bs-3, Bs-5, Bs-6, Bs-7, Bs-8, Bs-9, Bs-10, Bs-11, Bs-12, Bs-13, Bs-16, Bs-20, Bs-21, Bs-22, Bs-23, Bs-24, Bs-37, Bs-40, Bs-41, Bs-44, Bs-47, Bs-48
SP2	25	Bs-1, Bs-4, Bs-14, Bs-15, Bs-17, Bs-18, Bs-19, Bs-25, Bs-26, Bs-27, Bs-28, Bs-29, Bs-30, Bs-31, Bs-32, Bs-33, Bs-34, Bs-35, Bs-36, Bs-38, Bs-39, Bs-42, Bs-43, Bs-45, Bs-46

#### 3.4.2 Population genetic structure and diversity analyses

Population genetic analyses using GenAlEx 6.5, PCoA, and DARwin consistently identified two genetically distinct lineages among the 48 *B. sorokiniana* isolates. AMOVA demonstrated that 29% of the total molecular variance was due to differences between the two subpopulations, while 71% was found within subpopulations. Both components were highly significant (*PhiPT* = 0.295, *p* < 0.001; *Nm* = 1.195), indicating moderate genetic differentiation and limited gene flow (Table 11). Genetic diversity indices showed that SP2 exhibited higher allelic richness and heterozygosity (*Na* = 11; *Ne* = 8.333; *I* = 2.238; *H* = 0.880; *uH* = 0.917) compared to SP1 (*Na* = 8; *Ne* = 7.118; *I* = 2.011; *H* = 0.860; *uH* = 0.900), suggesting greater genetic diversity within SP2 (Table 12). PCoA separated the isolates into two distinct clusters, with the first two axes accounting for 33.41% of the total variation, consistent with the STRUCTURE-based grouping (Figure 5b). The phylogenetic tree generated by the Weighted Neighbour-Joining method in DARwin 6 also divided all isolates into two well-supported clades (Figure 5c), corroborating the STRUCTURE and PCoA results.

**Table 11:** Analysis of molecular variance (AMOVA) based on 48 *Bipolaris sorokiniana* isolates.

**Source**	**Df (Degrees of freedom)**	**SS (Sum of squares)**	**MS (Mean of squares)**	**Estimated variance**	**% (Percentage)**
Among populations	1	59.527	59.527	2.259	29%
Within population	46	248.431	5.401	5.401	71%
Total	47	307.958	64.928	7.660	100%
*PhiPT*	0.295				
*Nm* (haploid)	1.195				

Probability, P (rand > = data), for PhiPT is based on a standard permutation across the full data set. PhiPT (Pair wise population distribution) = AP/(WP + AP) = AP/TOT. Nm (Haploid) = [(1 / PhiPT) - 1] / 2.

**Table 12:** Mean values of genetic diversity parameters.

**Pop**	***N***	***Na***	***Ne***	***I***	***H***	***uH***
SP1	23	8	7.118	2.011	0.860	0.900
SP2	25	11	8.333	2.238	0.880	0.917
Mean	23.500	9.500	7.725	2.125	0.870	0.909
SE	1.500	1.500	0.608	0.113	0.010	0.008

Na- number of different alleles, Ne- number of effective alleles, I- Shannon’s information Index, H-Heterozygosity, uH- Unbiased heterozygosity, SP1- subpopulation-1, SP2- subpopulation-2, SE- standard error.

## 4 Discussion

*Bipolaris sorokiniana* populations responsible for spot blotch in Indian wheat display considerable morphological, cultural, pathogenic, and molecular heterogeneity. This diversity organized into at least two partially differentiated genetic lineages, each defined by unique diversity parameters (Kashyap et al., 2022; Roy et al., 2023). These findings are consistent with recent regional and phylogeographic studies and advance current understanding by integrating quantitative analyses of cultural and morphological characteristics, standardized pathogenicity assays on both susceptible and resistant wheat cultivars, and SSR-based population genetic analyses across a broad, spatially diverse set of Indian isolates (Chauhan et al., 2017; Sharma et al., 2022; Chakraborty et al., 2024). By integrating cultural characterization, pathogenicity assays on contrasting wheat genotypes, and SSR-based genetic analyses, this study provides baseline insights into pathogen variability across geographically diverse Indian wheat-growing regions. However, the findings interpreted within the methodological limitations of the study, particularly the use of limited molecular loci for species confirmation and the moderate resolution provided by SSR markers.

### 4.1 Morpho-cultural diversity and epidemiology

The observed variation in radial growth rates on PDA medium (2.50–8.45 mm day^−1^) and the clear stratification of isolates into discrete cultural classes based on colony color, texture, margin, elevation, and exudation demonstrate substantial phenotypic plasticity in *B. sorokiniana* under standardized *in vitro* conditions. This level of plasticity is comparable to, or greater than, that reported for Indian *B. sorokiniana* populations associated with spot blotch and black point diseases, where colony morphology and pigmentation also exhibited extensive variation without clear geographic patterns (Chauhan et al., 2017). The classification of isolates into five morpho-cultural clusters using Ward’s method, together with consistent divergence in radial growth patterns across multiple time points, suggests that these cultural phenotypes represent stable strain characteristics rather than transient responses to micro-environmental changes. This interpretation firmly supports the results from URP-PCR and RAPD-based variability studies, where distinct phenotypic groups were supported by multilocus marker data (Baturo-Cieśniewska, 2011; Mann et al., 2014).

The strong positive correlations among conidial length, breadth, and septation build upon previous findings that *B. sorokiniana* populations retain species-diagnostic pigmentation and general conidial architecture, while displaying significant variation in size classes and septation ranges across regions and hosts (Mann et al., 2014; Chauhan et al., 2017). The absence of strong associations between radial growth and qualitative cultural characteristics, such as colour classes, margin regularity, and exudation, indicates that these colony traits are unreliable as indicators of ecological fitness or virulence. Instead, these features likely reflect complex regulatory or metabolic differences that are not directly associated with aggressiveness, as observed in *B. sorokiniana* populations from barley and wheat in Europe and South America (Baturo-Cieśniewska, 2011; Mann et al., 2014). From an epidemiological perspective, larger and highly septate conidia, as seen in isolate Bs-3, may confer increased inoculum efficiency, improved survival on residues and phyllo planes and enhanced dispersal. These factors can increase epidemic potential under the warm, humid conditions typical of the Eastern Gangetic Plains and other high-risk environments (Al-Sadi, 2021; Roy et al., 2023). Although significant variation in radial growth observed among isolates, this variation did not correspond strongly with molecular subpopulation structure. This suggests that growth-related traits are likely to govern by general physiological factors rather than adaptive virulence-associated mechanisms. In contrast, pathogenic traits showed a clearer association with population structure, indicating that selective pressures related to host interaction play a more prominent role in shaping virulence than basic growth characteristics. Additional physiological and ecological studies would be required to validate these associations. Multilocus sequence databased population structure and robust markers are required for further validation (Baturo-Cieśniewska, 2011; Mann et al., 2014).

### 4.2 Pathogenic variability, virulence, and differential responses in wheat genotypes

The substantial pathogenic variability among isolates, as determined by incubation period, lesion size, infection frequency, lesion coverage, disease score, and percent disease index on both ‘Sonalika’ and ‘Chirya-3’, supports the multiple independent reports indicating that *B. sorokiniana* populations in South Asia display a continuum of aggressiveness rather than discrete pathotypes (Sultana et al., 2018; Roy et al., 2023). The consistent identification of Bs-3 as a highly virulent isolate, exhibiting the shortest incubation period, largest lesions, maximum lesion coverage, and highest disease score, corroborates recent findings in which a small subset of isolates in eastern India and Bangladesh were classified as “hyper-aggressive” and recommended as reference inoculum for differential screening and resistance phenotyping (Sultana et al., 2018; Chakraborty et al., 2024). These hyper-aggressive isolates may exhibit enhanced infection efficiency and accelerated tissue colonization kinetics, potentially linked to increased phytotoxin production and optimized secretomes, as indicated by comparative genomic analyses that reveal extensive variation in predicted effectors, secondary metabolite clusters, and CAZyme profiles across *B. sorokiniana* genomes (Leng et al., 2024; Shukla et al., 2025).

The strong negative correlation between incubation period and disease score (*r* ≈ −0.87), along with similarly negative associations with lesion length, breadth, size, coverage, and infection frequency, reinforces previous conclusions that rapid symptom onset is a key determinant of virulence in *B. sorokiniana* and related leaf blotch pathogens (Sultana et al., 2018; Sharma et al., 2022). Additionally, the highly significant positive correlations among lesion traits and disease severity are consistent with findings from leaf blotch studies in Nepal and Bangladesh, where isolates with shorter latent periods and larger necrotic lesions caused greater yield losses in susceptible wheat, particularly under high temperature and humidity in the Eastern Gangetic Plains (Sultana et al., 2018; Al-Sadi, 2021). Hierarchical clustering that grouped Bs-3, Bs-5, Bs-16, Bs-21, Bs-24, Bs-30, Bs-41, and Bs-44 into the most virulent assemblage demonstrates the existence of a distinct “high-aggressiveness” subset within the broader population. This finding supports the earlier reports that a minority of isolates can drive much of the epidemic intensity in hotspot environments and should be on priority for screening (Roy et al., 2023; Chakraborty et al., 2024).

The contrasting responses of ‘Sonalika’ and ‘Chirya-3’ observed in this study are consistent with their established roles as susceptible and resistant checks, respectively, in spot blotch nurseries across the Indo-Gangetic Plains. ‘Chirya-3’ has consistently exhibited partial, quantitative resistance (Roy et al., 2023). The observed reductions in lesion expansion, infection frequency, and percent disease index (PDI) in ‘Chirya-3’ suggest the presence of durable, polygenic resistance mechanisms. These mechanisms likely complement the major and minor quantitative trait loci (QTL) for spot blotch resistance identified in CIMMYT and Indian germplasm, including loci on chromosomes 2B, 5B, and 7B (Tomar et al., 2021; Roy et al., 2023). Given the extensive pathogenic variation and the dynamic nature of *B. sorokiniana* populations in warm wheat environments, breeding programs should pyramid multiple QTL conferring adult-plant and partial resistance. This approach would buffer against the adaptive potential of the pathogen and reduce the risk of resistance breakdown (Sharma et al., 2022; Roy et al., 2023; Alkan et al., 2026). Pathogenic variability in *B. sorokiniana* is host-dependent phenomenon, where the expression of virulence varies significantly across wheat cultivars. This variability reflects complex host–pathogen interactions governed by both pathogen aggressiveness and host resistance mechanisms. The significant cultivar × isolate interaction observed in the present study supports the existence of variety-specific pathogenic responses, reported in recent studies (Alkan et al., 2022; Alkan et al., 2026). Such interactions highlight the importance of evaluating pathogen populations across multiple host genotypes to understand disease dynamics and to develop durable resistance strategies.

### 4.3 Molecular diversity and population structure in a broader regional context

SSR-based genotyping of 48 isolates across 28 loci revealed moderate to high genetic diversity, with a mean gene diversity of approximately 0.30 and an average PIC value of approximately 0.24. The majority of markers were polymorphic, aligning with previous studies employing larger SSR, URP-PCR, or AFLP marker panels, which also reported substantial allelic variation within and among *B. sorokiniana* populations (Mann et al., 2014; Sharma and Yogesh, 2015; Kashyap et al., 2022). Prior URP- and ISSR-based investigations indicated that *B. sorokiniana* populations generally lack strong clonal structure and instead exhibit complex multilocus genotypes, suggesting frequent recombination or elevated mutation rates. The current results, which reveal considerable allelic diversity within both subpopulations, further support the presence of a recombining or highly diverse reproductive system (Baturo-Cieśniewska, 2011; Mann et al., 2014). The identification of highly informative SSRs, including BS-24, BS-28, BS-11, and BS-39, with high diversity and PIC values, offers a practical subset of markers for future monitoring of temporal and spatial changes in population structure. These loci can be combined with recently developed gene-targeted and diagnostic markers to investigate virulence-associated diversity (Kashyap et al., 2022; Kashyap et al., 2023).

Population structure analyses using STRUCTURE, PCoA, and weighted neighbor joining consistently supported a predominant *K* = 2 model, dividing the isolates into two genetic subpopulations (SP1 and SP2) with moderate differentiation (*PhiPT* ≈ 0.30; *Nm* ≈ 1.2). This finding aligns with ITS haplotype analyses that identified multiple major lineages within Indian and global *B. sorokiniana* populations (Kashyap et al., 2022; Sharma et al., 2022). AMOVA results indicated that 29% of the total molecular variance was attributable to differences among these subpopulations, while 71% was within them. This distribution is consistent with earlier AFLP- and URP-based studies, which found that most diversity exists within local populations, suggesting extensive gene flow facilitated by seed movement, residue carryover, and long-distance conidial dispersal (Mann et al., 2014; Sharma and Yogesh, 2015). The greater allelic richness and heterozygosity observed in SP2 (*Na* = 11; *Ne* = 8.333; *I* = 2.238; *He* = 0.880; *uHe* = 0.917) compared to SP1 suggest that SP2 may represent a more diverse or older lineage, or one exposed to stronger or more heterogeneous selection pressures in warm, humid, high-intensity wheat–rice systems. This interpretation is supported by reports that populations in the Eastern Gangetic Plains exhibit particularly high genetic and pathogenic diversity (Kashyap et al., 2022; Roy et al., 2023). Integrating SSR-defined structure with ITS haplotypes and whole-genome sequencing will be essential to determine whether SP1 and SP2 differ systematically in effector repertoires, toxin biosynthetic clusters, and CAZyme complements, as comprehensive genomic analyses have documented extensive variability in virulence-associated gene families in *B. sorokiniana* (Leng et al., 2024; Shukla et al., 2025). The SSR-based population structure revealed two distinct subpopulations (SP1 and SP2), which differed significantly in pathogenic behavior. Notably, isolates belonging to SP2 exhibited higher virulence, suggesting that genetic structuring within the population is functionally relevant. Although SSR markers primarily capture neutral genetic variation, the observed association between subpopulation grouping and pathogenicity indicates that these genetic clusters may reflect underlying divergence in adaptive traits. Recent advances in molecular plant pathology have demonstrated that pathogenic variability in *B. sorokiniana* is found to be linked with differential expression of virulence-associated genes, including effectors and enzymes involved in host colonization (Özer et al., 2020; Leng et al., 2024, Basak et al., 2024). In this context, SP2 isolates exhibit higher aggressiveness due to enhanced expression or diversification of virulence factors. Furthermore, the partial disconnect between molecular clustering and certain pathogenic traits suggests that specific functional loci under selection—rather than genome-wide neutral variation govern virulence. This observation aligns with current models of host–pathogen co-evolution, in which host resistance imposes selective pressures that drive the diversification of key pathogenicity determinants. The observed clustering patterns do not necessarily reflect functional divergence or evolutionary lineages. Similarly, while some differences in pathogenicity observed between isolates belonging to different clusters, these associations remain correlative. The present study did not evaluate virulence genes, effector diversity, or functional genomic variation. Therefore, it would be inappropriate to infer direct genetic mechanisms underlying aggressiveness based solely on SSR clustering. Thus, integrating population structure with pathogenic variability, as demonstrated in this study, provides an important bridge between classical phenotypic analysis and emerging molecular insights into host–pathogen interactions by using SSR markers. Although ITS-based identification used in present study, the use of additional loci such as *tef1* or *gapdh* could provide higher resolution for distinguishing closely related or cryptic species. Future studies using higher-resolution approaches such as multilocus sequence analysis, SNP genotyping, genome-wide association studies, or whole-genome sequencing would provide stronger insights into population structure and virulence evolution.

### 4.4 Integrating phenotype, genotype, and environment for durable management

The limited congruence among morpho-cultural clusters, pathogenicity groups, and SSR-based genetic structure observed in this study indicates that *B. sorokiniana* populations are influenced by environmental adaptation, host selection, and demographic processes. Neutral markers account for only a portion of the observed variation in virulence (Mann et al., 2014; Sultana et al., 2018). Highly virulent isolates, such as Bs-3, occur within specific pathogenicity groups but are not confined to a single molecular subpopulation. This finding aligns with previous studies showing that aggressiveness is often independent of neutral marker lineages in *B. sorokiniana* and other necrotrophs, emphasizing the importance of incorporating functional markers and effector-targeted assays in population studies (Baturo-Cieśniewska, 2011; Shukla et al., 2025). The substantial and coordinated variation among lesion traits, along with the clear separation of isolates into low- and high-virulence clusters, suggests that a concise set of composite pathogenicity metrics, including incubation period, lesion size, infection frequency, and PDI, can effectively distinguish aggressiveness for epidemiological modelling and germplasm screening (Sultana et al., 2018; Roy et al., 2023).

Rapid climatic changes and the documented expansion of spot blotch into traditionally cooler, low-disease regions, such as the North Western Plain Zone, highlight the necessity for robust pathogen surveillance and regular updates of reference isolates and screening protocols (Al-Sadi, 2021; Sharma et al., 2022). The identification of Bs-3, a highly virulent, morphologically distinctive, and genetically characterized isolate from Manikchak (West Bengal), serves as a valuable resource for standardized, high-stringency screening of breeding lines and for comprehensive host–pathogen interaction studies. Such studies may utilize transcriptomics, metabolomics, and effectoromics to elucidate resistance mechanisms and pathogen adaptation (Roy et al., 2023; Chakraborty et al., 2024). The high level of variability observed in the present study is consistent with previous large-scale surveys reporting widespread distribution and heterogeneous populations of *B. sorokiniana* across diverse wheat-growing regions. This supports the hypothesis that environmental adaptation and genetic diversity contribute significantly to disease dynamics. The management practices include deploying resistant cultivars in mixtures and rotations, seed health management, residue management, and optimized agronomy to reduce inoculum pressure and limit the selection of highly aggressive genotypes (Al-Sadi, 2021; Roy et al., 2023). The morpho-cultural, pathogenic, and molecular findings presented in this study advance the understanding of *B. sorokiniana,* population biology in India and provide a foundation for developing durable, climate-resilient strategies to control spot blotch in wheat. Some association observed between molecular clustering and pathogenic behavior, particularly with relatively aggressive isolates occurring more frequently within one subpopulation. However, these findings remain associative rather than causal. The present study did not investigate effector genes, toxin biosynthesis pathways, or other functional determinants of virulence. Therefore, direct links between genetic structure and pathogenicity cannot established based on the current dataset. Future studies using higher-resolution approaches such as multilocus sequence typing, SNP genotyping, genome-wide association studies, and whole-genome sequencing would provide stronger insights into population differentiation and virulence evolution.

## 5 Conclusion

This study provides information on morpho-cultural, pathogenic, and molecular variability among Indian populations of *B. sorokiniana*, the causal agent of spot blotch in wheat. The results demonstrate a highly heterogeneous and dynamically structured pathogen population, which has significant implications for epidemiology and breeding strategies. Persistent variation in cultural growth patterns and conidial morphology, together with strong positive correlations between conidial size and septation, indicate coordinated phenotypic differentiation linked to survival, dispersal, and inoculum efficiency, rather than transient environmental influences. Pathogenicity assays conducted on diverse wheat genotypes revealed a continuous variation in aggressiveness among isolates. Rapid infection onset and lesion expansion emerged as primary reasons of virulence, as evidenced by strong negative associations between incubation period and disease severity, and positive correlations among lesion-related traits. The repeated identification of Bs-3 as highly aggressive isolate across all disease parameters highlights the presence of hypervirulent genotypes in Indian populations and supports its application as a reference isolate for resistance screening and host–pathogen interaction studies. SSR-based genotyping separated the population into two moderately differentiated yet partially admixed genetic lineages, with the majority of molecular variation occurring within subpopulations. SSR analysis suggested genetic diversity within the population; however, the moderate level of marker polymorphism limits strong conclusions regarding population structure. Likewise, the observed relationship between molecular clustering and pathogenicity interpreted as associative and need further validation. The limited correspondence between neutral genetic structure and pathogenicity clusters indicates that virulence evolution in *B. sorokiniana* is not strictly lineage-dependent, but determined by host selection and environmental pressures on specific functional loci. Overall, the study contributes useful baseline information for future resistance breeding and disease management programs. Further research using multilocus phylogenetic, high-resolution genomics and functional studies are necessary to understand pathogen evolution and virulence mechanisms. Collectively, these findings suggest that Indian *B. sorokiniana* populations exhibit high adaptive potential in warm, humid agroecosystems. Effective management of spot blotch will require continuous pathogen surveillance, the use of well-characterized aggressive isolates in phenotyping, and the deployment of wheat cultivars with pyramided quantitative resistance to limit pathogen diversification under changing climatic conditions.

## Data Availability

The original contributions presented in the study are included in the article/Supplementary material, further inquiries can be directed to the corresponding author/s.
