# Supplementary material for: Morpho-cultural, pathogenic, and molecular diversity, and population structure analysis of Bipolaris sorokiniana causing spot blotch disease in wheat
Source: Front Microbiol. 2026 Jun 17;17:1811921. doi: 10.3389/fmicb.2026.1811921 (PMC13319100; doi:10.3389/fmicb.2026.1811921)
Supplement: Supplementary file 1 [file Table_1.docx]

**Supplementary Table 1: List of Bipolaris sorokiniana isolates used during the study**

| **Sr. No.** | **Isolates** | **Location** | **State** | **Latitude (° N)** | **Longitude (° E)** |
| --- | --- | --- | --- | --- | --- |
|  | BS-1 | Ambala | Haryana | 30.3752 ° N | 76.7821° E |
|  | BS-2 | Shamli | Uttar Pradesh | 29.4502° N | 77.3172° E |
|  | Bs-3 | Manikchak, Malda | West Bengal | 25.0777° N | 87.9003° E |
|  | Bs-4 | Uchani, Karnal | Haryana | 29.6876° N | 77.0020° E |
|  | Bs-5 | Uncha Samana, Karnal | Haryana | 29.6214° N | 76.9981° E |
|  | Bs-6 | Majra Roran, Karnal | Haryana | 29.7060° N | 76.7254° E |
|  | Bs-7 | Rusulpur Kalan, Karnal | Haryana | 29.6686° N | 77.0651° E |
|  | Bs-8 | Nalwi Khurd, Karnal | Haryana | 29.6942° N | 77.0939° E |
|  | Bs-9 | Dhanuauda, Jind | Haryana | 29.5301° N | 75.9957° E |
|  | Bs-10 | Anjanthali, Karnal | Haryana | 29.8344° N | 76.8879° E |
|  | Bs-11 | Barthal, Karnal | Haryana | 29.8670° N | 76.8735° E |
|  | BS-12 | Godhana, Chandauli | Uttar Pradesh | 25.2618° N | 83.3991° E |
|  | Bs-13 | Mohammadpur, Ballia | Uttar Pradesh | 25.4159° N | 83.5598° E |
|  | BS-14 | Futiya, Chandauli | Uttar Pradesh | 25.2561° N | 83.2780° E |
|  | BS-15 | Khajura, Jaunpur | Uttar Pradesh | 25.4663° N | 84.9831° E |
|  | Bs-16 | Rampur urf Akabarpur, Chandauli | Uttar Pradesh | 28.8029° N | 79.0254° E |
|  | BS-17 | Basani, Chandauli | Uttar Pradesh | 25.2778° N | 83.1890° E |
|  | BS-18 | Sakaldiha, Chandauli | Uttar Pradesh | 25.3502° N | 83.2577° E |
|  | BS-19 | Kurhua, Varanasi | Uttar Pradesh | 25.2232° N | 82.9598° E |
|  | BS-20 | Rasra, Ballia | Uttar Pradesh | 25.8591° N | 83.8585° E |
|  | BS-21 | Bahdura, Ballia | Uttar Pradesh | 26.0202° N | 84.1359° E |
|  | BS-22 | Bhakari, Jaunpur | Uttar Pradesh | 25.7927° N | 82.5756° E |
|  | BS-23 | Khanwa, Varanasi | Uttar Pradesh | 25.2345° N | 82.9433° E |
|  | BS-24 | Tarapur, Varanasi | Uttar Pradesh | 25.2213° N | 82.9774° E |
|  | BS-25 | Pratappur, Varanasi | Uttar Pradesh | 25.2795° N | 82.7965° E |
|  | Bs-26 | Tiuri, Sant Ravidas Nagar | Uttar Pradesh | 25.2591° N | 82.5965° E |
|  | Bs-27 | Natawa, Sant Ravidas Nagar | Uttar Pradesh | 25.2591° N | 82.5965° E |
|  | Bs-28 | Holpur, Sant Ravidas Nagar | Uttar Pradesh | 25.2924° N | 82.4058° E |
|  | Bs-29 | Vajja Patti, Sant Ravidas Nagar | Uttar Pradesh | 25.2881° N | 82.3412° E |
|  | Bs-30 | Sahasepur Harchahar Patti,  Sant Ravidas Nagar | Uttar Pradesh | 25.2468° N | 82.5504° E |
|  | Bs-31 | Bhiskuri, Mirzapur | Uttar Pradesh | 25.0928° N | 82.5976° E |
|  | Bs-32 | Parari, Mirzapur | Uttar Pradesh | 25.0946° N | 82.7507° E |
|  | Bs-33 | Bakiabad, Mirzapur | Uttar Pradesh | 25.1129° N | 82.9031° E |
|  | BS-34 | Khanoda, Kaithal | Haryana | 29.8386° N | 76.5387° E |
|  | BS-35 | Geong, Kaithal | Haryana | 30.0379° N | 76.7853° E |
|  | Bs-36 | Pundri, Kaithal | Haryana | 29.7621° N | 76.5546° E |
|  | Bs-37 | Mirzapur, Kurukshetra | Haryana | 29.9548° N | 76.7931° E |
|  | Bs-38 | Nilokheri, Karnal | Haryana | 29.8399° N | 76.9317° E |
|  | Bs-39 | Kachhwa, Karnal | Haryana | 29.7274° N | 76.8872° E |
|  | Bs-40 | Kalayat, Kaithal | Haryana | 29.6765° N | 76.2508° E |
|  | Bs-41 | Narvana, Jind | Haryana | 29.5960° N | 76.1150° E |
|  | Bs-42 | CCSHAU Hisar, Research Farm | Haryana | 29.1504° N | 75.7057° E |
|  | Bs-43 | Keorak, Kaithal | Haryana | 29.8698° N | 76.4708° E |
|  | Bs-44 | Sabour, Bhagalpur | Bihar | 25.2442° N | 87.0452° E |
|  | Bs-45 | Khanpur, Samastipur | Bihar | 25.8673° N | 85.9268° E |
|  | Bs-46 | Pundibari, Cooch Behar | West Bengal | 26.4121° N | 89.3843° E |
|  | Bs-47 | Uttar Dinajpur | West Bengal | 25.9810° N | 88.0510° E |
|  | Bs-48 | Umarsar, Shahjahanpur | Uttar Pradesh | 28.5252° N | 79.7611° E |
|  | Bs-49 | Kanakor, Pilibhit | Uttar Pradesh | 28.5379° N | 79.7448° E |
|  | Bs-50 | Andrayan, Pilibhit | Uttar Pradesh | 28.6818° N | 79.7523° E |
|  | Bs-51 | RPCAU Research Farm, Pusa | Bihar | 25.9845° N | 85.6742° E |
